# RBMS1-mediates the biogenesis of circNFIB promotes perineural invasion of pancreatic ductal adenocarcinoma via the L1CAM/MAPK pathway

**DOI:** 10.7150/thno.112753

**Published:** 2025-08-16

**Authors:** Zhuo Wu, Zhou Fang, Liangtang Zeng, Dingwen Zhang, Yu Zhou, Rufu Chen

**Affiliations:** Department of Pancreatic Surgery, Department of General Surgery, Guangdong Provincial People's Hospital (Guangdong Academy of Medical Sciences), Southern Medical University, Guangzhou, Guangdong Province, China.

**Keywords:** pancreatic ductal adenocarcinoma, perineural invasion, circNFIB, L1CAM, RBMS1

## Abstract

**Background:** Circular RNAs (circRNAs) play a key regulatory role in various functional characteristics of pancreatic ductal adenocarcinoma (PDAC). However, the mechanisms underlying circRNA's involvement in the occurrence of perineural invasion (PNI) in PDAC remain unclear and require further investigation.

**Methods:** Through circRNA sequencing, we identified the circNFIB (hsa_circ_0086376) that is highly associated with PNI in PDAC tissues. We then evaluated the promoting effect of circNFIB on PNI using various assays, including the Matrigel/dorsal root ganglia (DRG) model, DRG-matrix assay, transwell assay, orthotopic xenograft model, and in vivo model of neural infiltration. The interaction mechanism between circNFIB and IGF2BP3, which enhances L1CAM mRNA stability, was explored using RNA pulldown, mass spectrometry, RNA Immunoprecipitation (RIP), and actinomycin D assays. Additionally, the role of RBMS1 in promoting the biogenesis of circNFIB was investigated using RIP and Western blotting.

**Results:** This study confirmed that circNFIB is significantly upregulated in PDAC samples and samples with high PNI. Both in vitro and in vivo experiments demonstrated its role in promoting PNI in PDAC. Mechanistically, circNFIB binds with IGF2BP3 in PDAC cells to enhance the stability of L1CAM mRNA, activating the ERK/MAPK signaling pathway, and facilitating PNI in PDAC. Additionally, we found that RBMS1 binds to the NFIB pre-mRNA and promotes the biogenesis of circNFIB. Finally, we verified circNFIB as a potential therapeutic target that can mitigate the anti-tumor effects of SCH772984 in vivo.

**Conclusion:** RBMS1-mediated circNFIB interacts with IGF2BP3 to stabilize L1CAM mRNA, thereby activating the ERK/MAPK signaling pathway and promoting PNI in PDAC. This study provides a novel perspective on the molecular mechanisms underlying PNI in PDAC and lays the theoretical foundation for circNFIB as a potential therapeutic target for PDAC.

## Introduction

Pancreatic ductal adenocarcinoma (PDAC) is a highly malignant tumor with a five-year survival rate of only 11% 1, 2. Due to the lack of effective early diagnostic methods, most PDAC cases are diagnosed at an advanced stage, making curative surgery difficult 3. Perineural invasion (PNI) is a key pathological feature of PDAC, with an incidence exceeding 100%, which is significantly higher than that of other solid tumors 4. PNI often leads to refractory pain in PDAC patients, hinders radical resection, and ultimately affects survival 5-7. Previous studies have shown that PNI is associated with abnormalities in non-coding RNAs, proteins, and genes within the tumor microenvironment 8. Therefore, elucidating the molecular mechanisms underlying the occurrence and progression of PNI in PDAC and identifying effective therapeutic targets holds significant clinical value.

Circular RNAs (circRNAs) are a class of non-coding RNAs generated by back-splicing of precursor mRNAs (pre-mRNAs) exons. Their unique structure, lacking a 5'N7-methylguanosine cap and a 3' polyadenylated tail, renders them highly stable and resistant to degradation 9. Studies have shown that circRNAs exert diverse biological functions, including acting as miRNA sponges, interacting with proteins, regulating transcription and splicing, and even translating short peptides, thereby playing a crucial role in tumor regulation 10. Due to their distinctive structure and biological functions, circRNAs have emerged as promising biomarkers and therapeutic targets for cancer 10, 11. Previous studies have demonstrated that circRNAs play a pivotal regulatory role in various functional aspects of PDAC 12, 13. However, the mechanisms by which circRNAs contribute to PNI in PDAC remain unclear and warrant further investigation.

The mitogen-activated protein kinase (MAPK) pathway is activated in various tumors and plays a crucial role in regulating tumor initiation, progression, and invasion 14. The MAPK pathway is primarily classified into three subfamilies: the extracellular signal-regulated kinase (ERK or MEK) pathway, the c-Jun N-terminal kinase (JNK) pathway, and the p38 MAPK pathway 15. As a key component of the MAPK signaling cascade, the ERK MAPK pathway is essential for regulating cell growth, proliferation, and survival 16. SCH772984, a selective ERK1/2 inhibitor, has demonstrated antitumor activity in pancreatic, colorectal, and melanoma cells 17. Given its pivotal role in tumor biology, the MAPK pathway holds great potential for cancer prevention and therapy. However, the interplay between circRNAs and the ERK/MAPK pathway remains largely unexplored and warrants further investigation.

L1CAM is a 200-220 kDa transmembrane glycoprotein belonging to the immunoglobulin (Ig) superfamily. It is highly expressed in PDAC, renal cell carcinoma, ovarian cancer, endometrial cancer, melanoma, and colorectal cancer 18-22. Previous studies have shown that L1CAM regulates tumor initiation and progression by activating key signaling pathways, including the STAT3, Wnt/β-catenin, and MAPK pathways 19, 23, 24. However, no studies have yet investigated the role of L1CAM in PNI in PDAC. Therefore, exploring its involvement in PDAC-associated PNI is of significant interest.

In this study, we identified the circular RNA, circNFIB (hsa_circ_0086376), as highly expressed in PDAC with high PNI and associated with poor prognosis. Through in vitro and in vivo functional experiments, we validated that circNFIB overexpression significantly enhances PNI in PDAC. Furthermore, we explored the underlying mechanisms by which circNFIB influences PNI. Mechanistically, circNFIB recruits the RNA-binding protein (RBP) IGF2BP3 to the 3' untranslated region (3'-UTR) of L1CAM mRNA, enhancing its stability and activating the ERK/MAPK signaling pathway, thereby promoting PNI in PDAC. Additionally, we investigated the biogenesis of circNFIB. We identified RBMS1 as a key regulator of circNFIB biogenesis in PDAC. RBMS1 facilitates back-splicing and accelerates circNFIB formation by binding to intron 2 [7051-7101 nt] and intron 6 [11801-11882 nt] of NFIB pre-mRNA. Finally, we demonstrated the therapeutic potential of targeting this pathway by treating PNI in an in vivo model with SCH772984, an ERK1/2 inhibitor, which exhibited promising efficacy. Our findings provide new insights into the molecular mechanisms driving PNI and suggest a potential therapeutic strategy for PDAC patients with PNI.

## Materials and Methods

### Clinical samples

This study included 96 pancreatic cancer patients who underwent surgical treatment at the Pancreatic Center of Guangdong Provincial People's Hospital. Surgical resection tissue samples and clinical data were collected from the hospital's medical record system. Follow-up information was confirmed through telephone interviews and death certificates. The study protocol was approved by the Ethics Committee of Guangdong Provincial People's Hospital (Approval No. KY2024-528), and informed consent was obtained from all participants.

### RNA Immunoprecipitation (RIP) assay

Firstly, 2×10⁷ PDAC cells were lysed using RIP lysis buffer (BersinBio, China). Subsequently, antibodies against IgG, AGO2, IGF2BP3, and RBMS1 were incubated with the cell lysates overnight at 4 °C. The following day, magnetic beads (BersinBio, China) were added to the antibody-lysate complexes and incubated for 3 h at 4 °C. The precipitated RNA samples were extracted, reverse-transcribed into cDNA, and analyzed using a qRT-PCR assay.

### Isolation of DRG cells

Four-week-old nude mice were euthanized following intraperitoneal injection of pentobarbital anesthesia. The spine was exposed and dissected along the longitudinal axis to extract the dorsal root ganglia (DRG). The DRG tissues were digested at 37 °C for 30 min with 1 ml of 0.2% type IV collagenase and 0.25% trypsin to facilitate dissociation. The reaction was then terminated by adding 2 ml of fetal bovine serum (FBS). The digested tissues were filtered through 100 µm and 40 µm cell strainers and subsequently cultured in DMEM medium. On the following day, 5 μM cytarabine was added to eliminate proliferating cells.

### Transwell assay

A total of 1×10⁵ isolated DRG cells were seeded into 24-well tissue culture plates. Subsequently, 5×10⁴ transfected PDAC cells were resuspended in 200 μl of serum-free medium and added to Transwell inserts (Corning, USA) pre-coated or uncoated with Matrigel. The inserts were then placed into the 24-well tissue culture plates for co-culture. After 12 h of incubation, the invaded cells were fixed with 4 % paraformaldehyde and stained with crystal violet solution (Beyotime, China). Images were captured using a microscope (Olympus, Tokyo, Japan), and migrated cells were quantified using ImageJ software.

### Matrigel/DRG Model

First, DRG were isolated from nude mice. A total of 20 μL Matrigel (BD Biosciences, USA) was added dropwise into a six-well plate, and the DRG was embedded within the Matrigel. Next, 2×10⁵ transfected PDAC cells were resuspended in 20 μL Matrigel and seeded at a distance of 2 mm from the DRG edge. After the Matrigel solidified, 2 mL of DMEM medium containing 10% FBS was added to each well for culture. After 7 days, the migration behavior of PDAC cells and DRG was evaluated. The migration distance of PDAC cells was defined as α, the migration distance of DRG cells as β, and the initial distance between PDAC cells and DRG as c. The invasion index (α/c) was used to assess the neurotropic behavior of PDAC cells, while the growth index (β/c) reflected the neural growth capacity 25.

### DRG-Matrix Assay

First, DRG were isolated from nude mice. A total of 5 μL Matrigel (BD Biosciences, USA) was added dropwise into a six-well plate, and the DRG was embedded within the Matrigel. After culturing in 2 ml DMEM medium containing 10% FBS for 3 days, 1×10⁵ GFP-labeled transfected PDAC cells were added to the wells. Three days later, the fluorescence signals of PDAC cells invading the Matrigel were quantified using ImageJ software to assess the invasive capability of PDAC cells.

### In Vivo Model of Neural Infiltration

Nude mice were anesthetized with inhaled pentobarbital, and the right sciatic nerve was exposed. A total of 1×10⁵ luciferase-labeled transfected PDAC cells were injected into the sciatic nerve, followed by suturing of the incision. Sciatic nerve function was assessed weekly using a nerve function scoring system 35. After 6 weeks, tumors were imaged using an IVIS imaging system. Sciatic nerve samples were then collected for H&E staining to evaluate the severity of PNI within the sciatic nerve.

### Statistical Analysis

Experimental data in this study were obtained from at least three independent experiments. Quantitative data are presented as mean ± standard deviation (SD). Parametric variables were analyzed using a two-tailed Student's t-test or one-way ANOVA, while non-parametric variables were evaluated using the chi-square test or Fisher's exact test. Overall survival (OS) was assessed using the Kaplan-Meier method, and independent prognostic factors were identified through a multivariate Cox proportional hazards model.

A *p* < 0.05 was considered statistically significant. Statistical analyses were performed using Graphpad Prism 8 (version 8.0.2; Graphpad Software, USA) and SPSS (version 27.0.0; IBM SPSS Statistics, USA).

## Results

### Identification and Expression of CircNFIB in PDAC

**Figure 1:**
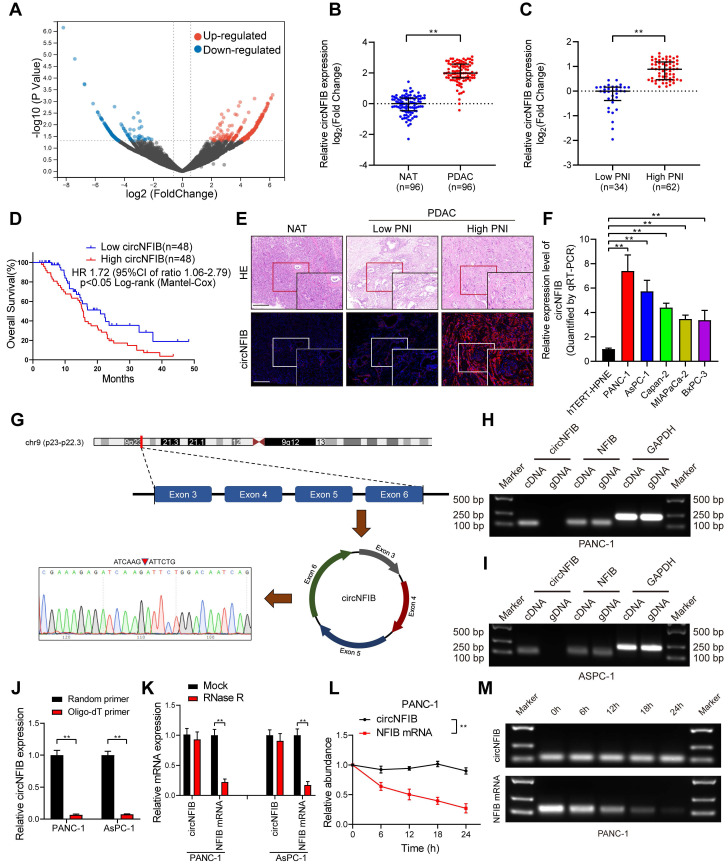
** Identification and Characterization of circNFIB in PDAC.** (A) Volcano plot of differentially expressed circRNAs. (B) qRT-PCR expression of circNFIB in PDAC tissues (n = 96) paired with NATs (n = 96). (C) qRT-PCR expression of circNFIB in PDAC tissues with low PNI (n = 34) and high PNI (n = 62). (D) Kaplan-Meier survival curve of overall survival (OS) in PDAC patients with low and high circNFIB expression levels. (E) Representative images of RNA fluorescence in situ hybridization (FISH) showing circNFIB upregulation in high PNI tissues compared to low PNI tissues. (F) qRT-PCR analysis of circNFIB expression in hTERT-HPNE, PANC-1, AsPC-1, Capan-2, MiaPaCa-2, and BxPC-3 cell lines. (G) Schematic illustration of circNFIB circularization and Sanger sequencing of the splice junction. (H, I) PCR analysis of circNFIB and NFIB in cDNA and gDNA of PANC-1 and AsPC-1 cells using GAPDH as a control. (J) qRT-PCR analysis of circNFIB expression using random primers or oligo-dT primers. (K) qRT-PCR analysis of circNFIB and NFIB mRNA expression in PANC-1 and AsPC-1 cells after RNase R treatment. (L, M) Stability assessment of circNFIB and NFIB mRNA in PANC-1 cells at designated time points using actinomycin D assay (L) and agarose gel electrophoresis (M). Statistical significance was assessed using the non-parametric Mann-Whitney U test for B and C, one-way ANOVA followed by Dunnett's test for F, and two-tailed Student's t-test for J, K, and L. Error bars represent SD from three independent experiments. *p < 0.05, **p < 0.01.

To identify differentially expressed circRNAs associated with PNI in PDAC, we performed circRNA sequencing on three PNI-positive and three PNI-negative PDAC tissue samples. The circRNA sequencing data are available from the Gene Expression Omnibus (GEO) under accession code GSE303688. To minimize bias caused by extreme expression in individual samples, only circRNAs detected in all samples were considered for ranking and further validation. Differential expression analysis was conducted using DESeq2, and circRNAs with a log2 fold change (log2FC) > 1.5 and a *p* < 0.05 were considered significantly upregulated (Figure 1A). The top 10 circRNAs meeting these criteria were selected for further experimental validation. To further validate our findings, we conducted analysis in a larger cohort of 96 PDAC patients. We found that circNFIB was significantly upregulated in both PDAC and PNI samples, and its expression was significantly associated with poor prognosis in these patients (Figure 1B-D). Additionally, univariate and multivariate Cox regression analyses identified circNFIB expression as an independent predictor of poor prognosis (Table 1, Table S1). RNA fluorescence in situ hybridization (FISH) analysis confirmed that the expression level of circNFIB was consistent with the above results (Figure 1E). We utilized qRT-PCR to measure the expression levels of circNFIB in five common PDAC cell lines (PANC-1, AsPC-1, MiaPaCa-2, BxPC-3, Capan-2). The results revealed that circNFIB was most highly expressed in the PANC-1 and AsPC-1 cell lines (Figure 1F). We also performed the DRG/Matrigel model in five pancreatic cancer cell lines following circNFIB overexpression. The results showed that circNFIB consistently promoted PNI across all cell lines; however, the most pronounced effects were observed in PANC-1 and AsPC-1 cells. Therefore, we selected these two cell lines for subsequent experiments (Figure S1A).

To identify the structural characteristics of circNFIB, we performed Sanger sequencing on the qRT-PCR products amplified using circNFIB-specific primers, and successfully located its back-splicing junction (Figure 1G). PCR analysis showed that circNFIB was detectable only in complementary DNA (cDNA), whereas NFIB was expressed in both cDNA and genomic DNA (gDNA), suggesting that circNFIB is formed through back-splicing rather than genomic rearrangement (Figure 1H-I). In reverse transcription experiments using random primers, circNFIB exhibited a higher expression level compared to the oligo-dT primer product, indicating the absence of a poly-A tail (Figure 1J). Additionally, treatment of qRT-PCR products with RNase R and actinomycin D demonstrated that circNFIB exhibited greater stability than linear NFIB (Figure 1K-M, Figure S1B-C). Taken together, these experiments provide evidence that circNFIB is a highly expressed, stable, and closed circular RNA in PDAC.

### CircNFIB Promotes PNI in PDAC Cells In Vitro

**Figure 2:**
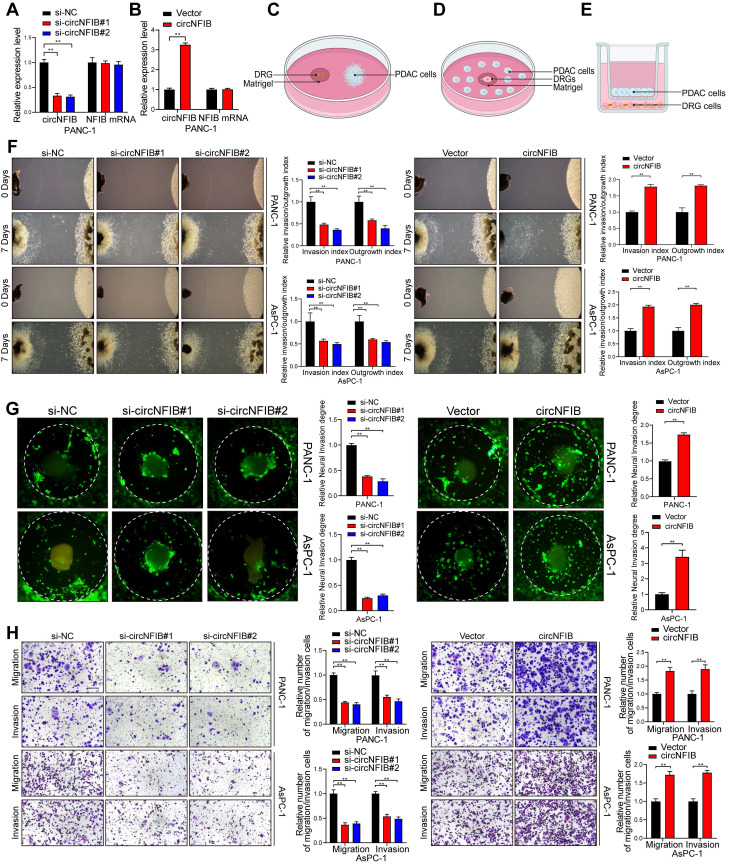
** CircNFIB Promotes PNI of PANC-1 and AsPC-1 Cells In Vitro.** (A, B) qRT-PCR analysis of circNFIB and NFIB expression levels in circNFIB knockdown (A), circNFIB overexpression (B), and corresponding control PANC-1 cells. (C-E) Schematic diagrams of the DRG Model, DRG Matrix, and Transwell assays. (F) Representative Matrigel/DRG images showing the neural invasion and growth capacity of PANC-1 and AsPC-1 cells following circNFIB knockdown or overexpression, with quantitative analysis. (G) Representative DRG Matrix images displaying the neural invasion ability of PANC-1 and AsPC-1 cells after circNFIB knockdown or overexpression, with quantification. (H) Representative images and quantification of Transwell migration and Matrigel invasion assays in PANC-1 and AsPC-1 cells upon circNFIB knockdown or overexpression. Scale bar = 100 μm. Statistical differences in A, F, G, and H were analyzed using one-way ANOVA followed by Dunnett's test, while B, F, G, and H were analyzed using a two-tailed Student's t-test. Error bars indicate SD from three independent experiments. *p < 0.05, **p < 0.01.

To further explore the role of circNFIB in promoting axon formation and PNI in PDAC cells, we first manipulated the expression of circNFIB in cell lines by transfecting small interfering RNA (siRNA) to knockdown or plasmids to overexpress circNFIB (Figure 2A-B, Figure S1D-E). We conducted in vitro invasion assays in pancreatic cancer cells, including Transwell and wound healing assays. The results indicated that circNFIB does not affect the general invasive capabilities of pancreatic cancer cells (Figure S1F-G).

To validate the impact of circNFIB on tumor-neuron interactions, we constructed a Matrigel/DRG model (Figure 2C). The extension of DRG axons was inhibited when co-cultured with PDAC cells in which circNFIB was knocked down, resulting in reduced neurotropism of PDAC cells. In contrast, co-culturing DRG with PDAC cells overexpressing circNFIB significantly enhanced axonal extension and notably increased the neurotropism of PDAC cells (Figure 2F, Figure S1H). Subsequently, to further confirm whether circNFIB expression is involved in PNI in PDAC cells, we performed DRG matrix assays (Figure 2D). The results showed that circNFIB knockdown significantly suppressed PDAC cell invasion towards DRG, while circNFIB overexpression significantly enhanced the invasive capacity of PDAC cells towards DRG (Figure 2G). We also conducted Transwell assays and found that knocking down circNFIB reduced tumor cell invasion of nerve cells, while overexpression of circNFIB significantly increased nerve cell invasion (Figure 2E and Figure 2H). These experimental results demonstrate that circNFIB significantly influences PDAC PNI in vitro.

### CircNFIB Promotes PNI in PDAC Cells In Vivo

**Figure 3:**
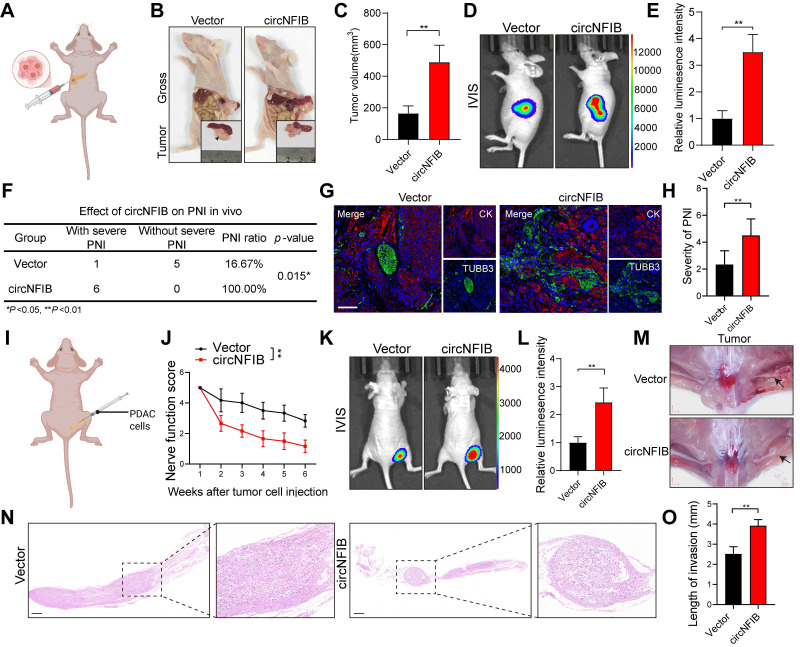
** CircNFIB Promotes PNI in PDAC In Vivo.** (A) Schematic diagram of the orthotopic xenograft tumor model in nude mice. (B-E) Representative harvested pancreatic tumors and statistical analysis in the orthotopic xenograft tumor model (B, C), along with IVIS imaging and corresponding quantification (D, E) (n = 6 nude mice per group). (F) PNI ratio in tumor tissues from the orthotopic xenograft tumor model. (G, H) Representative images of CK and TUBB3 multiplex immunohistochemical staining in the orthotopic xenograft tumor model. Scale bar = 100 μm. (I) Schematic diagram of the in vivo neural invasion model in nude mice. (J) Neurological function scores of PANC-1 tumor-bearing nude mice. (K-O) IVIS imaging and statistical analysis of the in vivo neural invasion model in nude mice (K, L), along with representative harvested tumors and H&E-stained images (M-O) (n = 6 nude mice per group). Statistical analysis for C, E, H, J, L, and O was performed using a two-tailed Student's t-test. χ² test was used for F. Statistical differences in J were analyzed using one-way ANOVA followed by Dunnett's test. Error bars represent SD from three independent experiments. *p < 0.05, **p < 0.01.

To investigate the in vivo role of circNFIB in PDAC PNI, we established an orthotopic pancreatic xenograft model using PANC-1 cells overexpressing circNFIB or a control vector (Figure 3A). Compared to the Vector group, the tumors in the circNFIB overexpression group exhibited significantly larger volumes, and the degree of PNI was markedly increased (Figure 3B-F). Subsequent multiplex immunohistochemistry (mIHC) analysis revealed that tumors overexpressing circNFIB showed a significant increase in nerve density, and the level of circNFIB correlated with the extent of PNI (Figure 3G-H).

Additionally, we injected PANC-1 cells overexpressing circNFIB or control cells into the sciatic nerve of mice (Figure 3I). After six weeks, nerve function scoring revealed significant hind limb dysfunction in the circNFIB overexpression group compared to the control group (Figure 3J). IVIS imaging and macroscopic examination further demonstrated that overexpression of circNFIB significantly enhanced the ability of PDAC cells to metastasize along the sciatic nerve, with tumors exhibiting larger volumes (Figure 3K-O). In summary, circNFIB promotes PDAC PNI in vivo, highlighting its potential role in driving tumor progression and nerve invasion.

### CircNFIB Promotes PNI in PDAC by Upregulating L1CAM Expression

**Figure 4:**
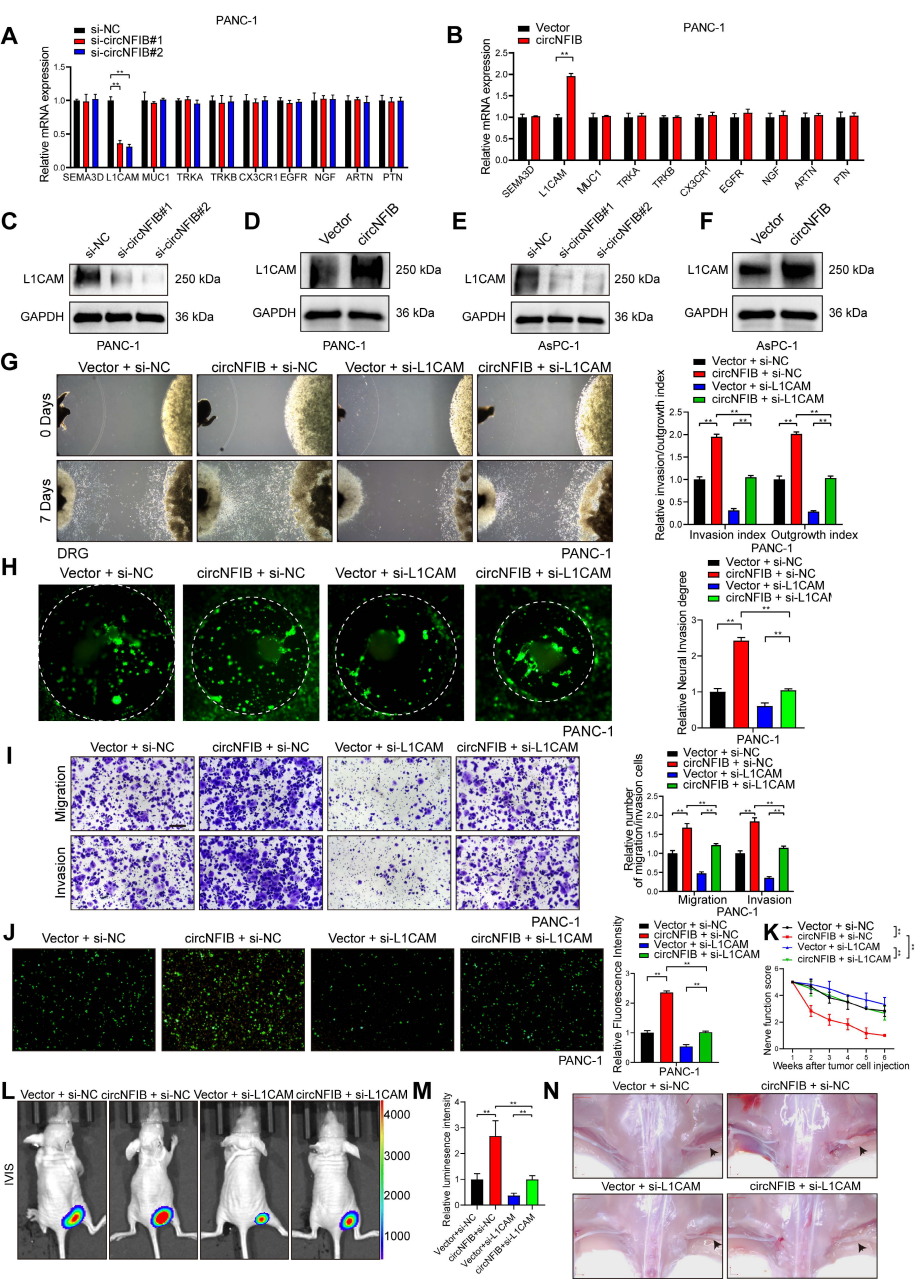
** CircNFIB Regulates L1CAM Expression in PDAC.** (A, B) qRT-PCR analysis of PNI-related gene expression levels following (A) circNFIB knockdown or (B) circNFIB overexpression. (C-F) Western blotting analysis of L1CAM expression levels in PDAC cells upon circNFIB knockdown (C, E) or circNFIB overexpression (D, F). (G) Representative Matrigel/DRG images and quantification of neural invasion and growth capacity in designated PANC-1 cells. (H) Representative DRG Matrix images and quantification of neural invasion ability in designated PANC-1 cells. (I) Representative images and quantification of Transwell migration and Matrigel invasion assays in designated PANC-1 cells. Scale bar = 100 μm. (J) Representative images and quantification of the neural adhesion assay using the indicated PANC-1 cells, showing the cell-nerve adhesion capacity. (K) Neurological function scores of PANC-1 tumor-bearing nude mice. (L-N) IVIS imaging and statistical analysis of the in vivo neural invasion model in nude mice (L, M), along with representative harvested tumor images (M) (n = 6 nude mice per group). Statistical differences for A, G, H, I, J, K, L, and M were analyzed using one-way ANOVA followed by Dunnett's test, while B was analyzed using a two-tailed Student's t-test. Error bars represent SD from three independent experiments. *p < 0.05, **p < 0.01.

Previous studies have identified various molecules that drive PNI in tumors 26. To further investigate the mechanism by which circNFIB regulates PNI in PDAC, we examined the expression levels of PNI-related molecules following the modulation of circNFIB expression using qRT-PCR. The results revealed that knockdown of circNFIB significantly decreased L1CAM expression, while overexpression of circNFIB led to a corresponding increase in L1CAM expression (Figure 4A-B). Western blot analysis confirmed these findings (Figure 4C-F, Figure S6A-D).

Next, we used the Matrigel/DRG model, DRG matrix assays, and Transwell assays to further investigate the functional role of L1CAM in circNFIB-mediated PNI. We found that knockdown of L1CAM significantly eliminated the enhanced PNI ability of PDAC cells overexpressing circNFIB (Figure 4G-I, Figure S2A-C). L1CAM is a neural cell adhesion molecule that has been increasingly recognized for its role in tumor progression, particularly in enhancing cancer cell motility, invasion, and perineural invasion. To further investigate this, we performed a Neural adhesion assay to evaluate whether L1CAM expression modulates the ability of tumor cells to adhere to nerves, thereby contributing to their invasive behavior. The results showed that knockdown of L1CAM significantly attenuated the enhanced neural adhesion induced by circNFIB overexpression (Figure 4J, Figure S2D). Furthermore, in vivo experiments involving injection of PANC-1 cells with L1CAM knockdown and circNFIB overexpression into the sciatic nerve of mice yielded similar results (Figure 4K-N). These findings suggest that circNFIB promotes PDAC PNI by regulating the expression of L1CAM.

### CircNFIB Enhances L1CAM mRNA Stability in PDAC Cells by Binding to IGF2BP3

Previous studies have shown that circRNAs exert their functions primarily as molecular sponges, by interacting with RNA-binding proteins, or through protein-coding roles 9. To clarify how circNFIB exerts its effects, we first conducted RNA nuclear-cytoplasmic fractionation and FISH experiments, confirming that circNFIB primarily localizes to the cytoplasm of PDAC cells (Figure S3A-D). Additionally, the circRNADb database (http://reprod.njmu.edu.cn/circrnadb/circRNADb.php) does not annotate an open reading frame (ORF) for circNFIB, suggesting that it may not have protein-coding potential. We also performed RIP assays, which showed no significant difference in the enrichment of circNFIB with Argonaute 2 (AGO2) antibodies compared to IgG controls, indicating that circNFIB does not function through miRNA sponging (Figure S3E). Therefore, we hypothesize that circNFIB may exert its effects through interaction with RNA-binding proteins.

**Figure 5:**
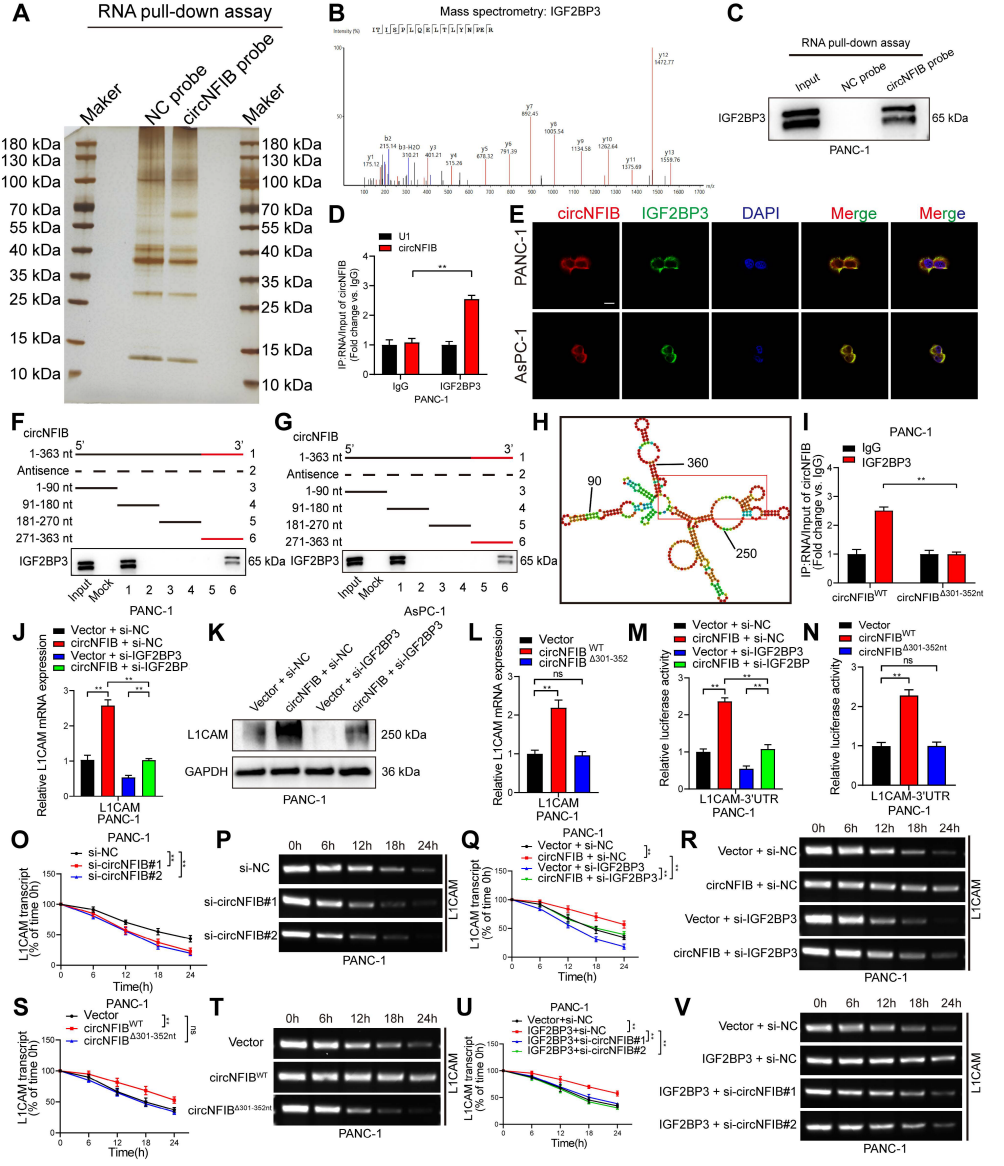
** CircNFIB Interacts with IGF2BP3 in PDAC Cells to Promote L1CAM mRNA Stability.** (A, B) Silver staining image of RNA pull-down assay using a circNFIB probe (A) and mass spectrometry analysis of IGF2BP3 protein (B). (C) Western blotting analysis of the interaction between circNFIB and IGF2BP3 in PANC-1 cells. (D) RIP assay confirming IGF2BP3 enrichment of circNFIB in PANC-1 cells. (E) Representative images of circNFIB and IGF2BP3 colocalization. Scale bar = 20 μm. (F, G) Deletion mapping analysis demonstrating that the 271-363 nt region of circNFIB is critical for its interaction with IGF2BP3. (H) Schematic representation of predicted IGF2BP3 binding sites on circNFIB. (I) RIP assay following deletion of the 301-352 nt region of circNFIB. (J, K) qRT-PCR (J) and Western blotting (K) analysis of L1CAM expression in designated PANC-1 cells. (L) qRT-PCR analysis of L1CAM expression in designated PANC-1 cells. (M, N) Luciferase reporter assay analyzing L1CAM mRNA 3'-UTR activity in designated PANC-1 cells. (O-V) Actinomycin D assay showing L1CAM mRNA stability and representative agarose gel electrophoresis images in designated PANC-1 cells. Statistical differences in J, L, M, N, O, Q, S, and U were analyzed using one-way ANOVA followed by Dunnett's test, while D and I were analyzed using a two-tailed Student's t-test. Error bars represent SD from three independent experiments. *p < 0.05, **p < 0.01.

To identify RNA-binding proteins that associate with circNFIB, we performed RNA pull-down assays using biotinylated probes targeting circNFIB. Silver staining revealed a distinct band in the circNFIB probe group, which was identified as Insulin-like growth factor II mRNA-binding protein 3 (IGF2BP3) through mass spectrometry (Figure 5A-B). Subsequent Western blot analysis of the RNA pull-down samples further confirmed the significant enrichment of IGF2BP3 (Figure 5C, Figure S3F). RIP experiments also demonstrated that circNFIB was significantly enriched in the IGF2BP3 antibody group compared to the IgG control group (Figure 5D). FISH and immunofluorescence analyses showed co-localization of circNFIB and IGF2BP3 in the cytoplasm of PDAC cells (Figure 5E).

To further investigate the binding site of IGF2BP3 on circNFIB, we performed a series of truncation experiments. The results showed that IGF2BP3 predominantly binds to the 271-363 nt region of circNFIB (Figure 5F-G). In silico prediction using the catRAPID tool (http://service.tartaglialab.com/page/catrapid_group) suggested that the 301-352 nt segment of circNFIB is a potential binding site for IGF2BP3 (Figure 5H). To validate the importance of this 301-352 nt region in the interaction between circNFIB and IGF2BP3, we mutated this segment and found that the RIP results showed a significant reduction in IGF2BP3 enrichment on circNFIB (Figure 5I).

Since L1CAM is a downstream target of circNFIB, we next investigated whether IGF2BP3 cooperates with circNFIB to regulate L1CAM expression. qRT-PCR and Western blot analyses showed that knockdown of IGF2BP3 significantly reversed the upregulation of L1CAM expression induced by circNFIB overexpression (Figure 5J-K, Figure S3G, Figure S6E, Figure S6L). Furthermore, compared to wild-type circNFIB, the mutated circNFIB (Δ301-352 nt) had no significant effect on L1CAM expression (Figure 5L). These results suggest that circNFIB regulates L1CAM expression by binding to IGF2BP3.

Previous studies have shown that IGF2BP3 can regulate mRNA stability by targeting the 3'-UTR of mRNAs 27, 28. Therefore, we investigated whether circNFIB can regulate L1CAM stability through its interaction with IGF2BP3. Dual-luciferase assays revealed that overexpression of circNFIB significantly increased luciferase activity in the 3'-UTR region of L1CAM mRNA, which was reversed upon IGF2BP3 knockdown (Figure 5M). Additionally, the mutated circNFIB (Δ301-352 nt) had no effect on the luciferase activity of L1CAM mRNA 3'-UTR (Figure 5N). These findings collectively support that the circNFIB/IGF2BP3 complex regulates L1CAM expression via its 3'-UTR.

Next, we assessed the impact of circNFIB on L1CAM mRNA stability using actinomycin D treatment. The results showed that knockdown of circNFIB significantly reduced the half-life of L1CAM mRNA (Figure 5O-P, Figure S3H-I). Conversely, overexpression of circNFIB extended the half-life of L1CAM mRNA, which was reversed upon IGF2BP3 knockdown (Figure 5Q-R, Figure S3J-K). Similarly, the mutated circNFIB (Δ301-352 nt) had no significant effect on L1CAM mRNA stability (Figure 5S-T, Figure S3L-M). Moreover, overexpression of IGF2BP3 increased the half-life of L1CAM mRNA, which was reversed by circNFIB knockdown (Figure 5U-V, Figure S3N-O). Additionally, RIP experiments demonstrated that IGF2BP3 significantly binds to the 3'-UTR of L1CAM mRNA (Figure S3P). Together, these results demonstrate that circNFIB stabilizes L1CAM mRNA in PDAC cells by binding to IGF2BP3 and interacting with the 3'-UTR of L1CAM.

### CircNFIB Regulates PDAC PNI via the ERK/MAPK Signaling Pathway

**Figure 6:**
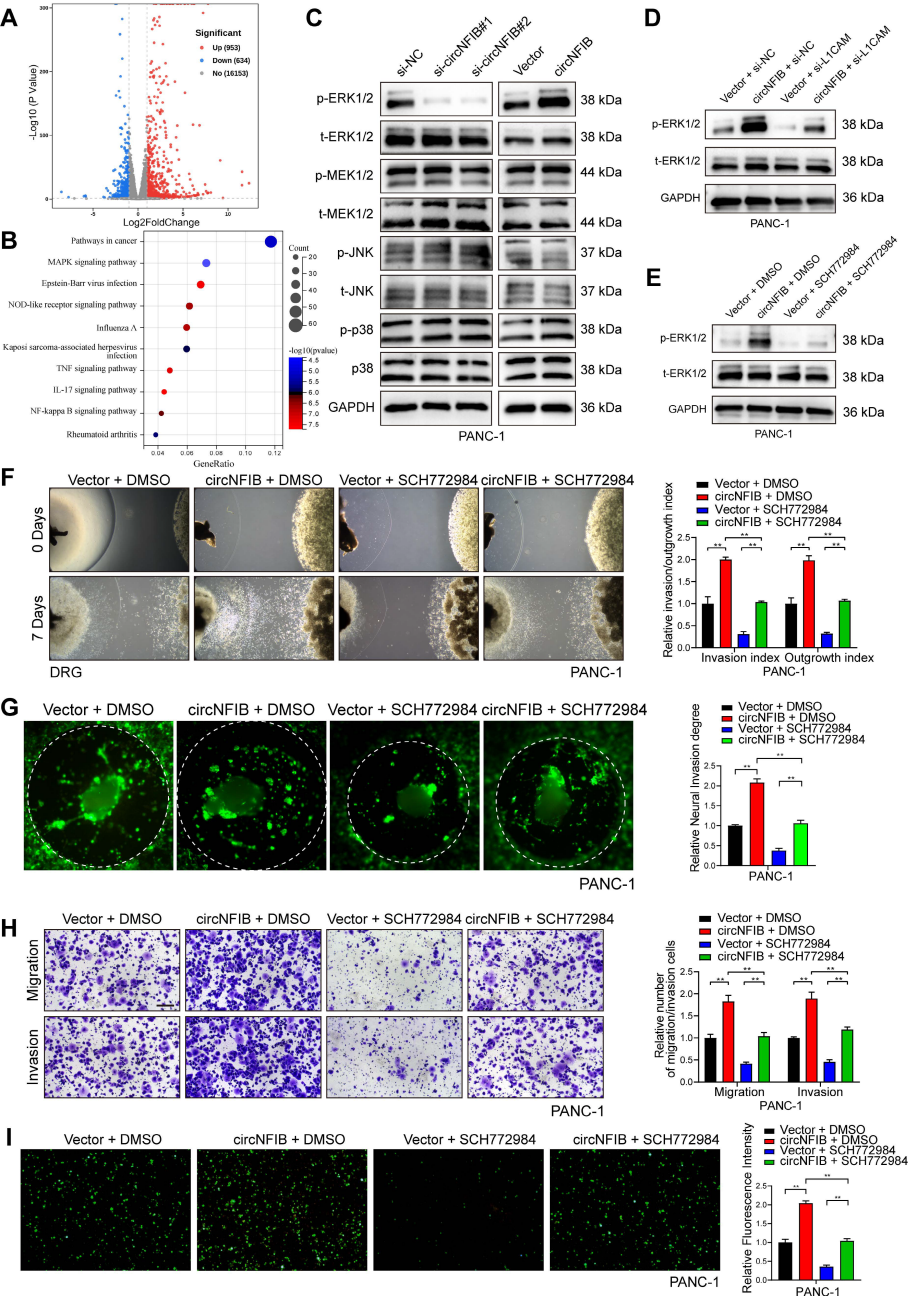
** CircNFIB Promotes PNI by Activating the ERK/MAPK Axis in PDAC.** (A) Volcano plot of differentially expressed genes between circNFIB-overexpressing and control groups. (B) KEGG pathway enrichment analysis of differentially expressed genes between circNFIB-overexpressing and control groups. (C) Western blotting analysis of MAPK signaling pathway-related genes in PDAC cells upon circNFIB knockdown or overexpression. (D) Western blot analysis of p-ERK and t-ERK levels in PANC-1 cells upon circNFIB and L1CAM modulation. (E) Western blot analysis of p-ERK and t-ERK levels in PANC-1 cells following SCH772984 treatment. (F) Representative Matrigel/DRG images and quantification of neural invasion and growth ability in designated PANC-1 cells. (G) Representative DRG Matrix images and quantification of neural invasion ability in designated PANC-1 cells. (H) Representative images and quantification of Transwell migration and Matrigel invasion assays in designated PANC-1 cells. Scale bar = 100 μm. (I) Representative images and quantification of the neural adhesion assay using the indicated PANC-1 cells, showing the cell-nerve adhesion capacity. Statistical differences in F, G, H, and I were analyzed using one-way ANOVA followed by Dunnett's test. Error bars represent SD from three independent experiments. *p < 0.05, **p < 0.01.

To further explore the potential mechanism by which circNFIB promotes PNI in PDAC cells, we performed high-throughput RNA-seq to identify differentially expressed genes (DEGs) in PANC-1 cells overexpressing circNFIB compared to control cells. The analysis revealed 1587 DEGs (*p* < 0.05 and log2 Fold Change >1). Kyoto Encyclopedia of Genes and Genomes (KEGG) pathway enrichment analysis indicated a significant activation of the MAPK signaling pathway (Figure 6A-B). Subsequently, we performed Western blotting, which showed that the phosphorylation of ERK was notably altered upon regulation of circNFIB expression (Figure 6C, Figure S4A, Figure S6F-G, Figure S6M-N). These findings suggest that circNFIB likely promotes PNI inPDAC through the ERK/MAPK signaling pathway.

Based on previous studies, MAPK is known to be a downstream pathway of L1CAM, which regulates cellular functions, including PNI 29, 30. Therefore, we hypothesized that circNFIB promotes PNI in PDAC by activating the MAPK pathway through L1CAM. To test this hypothesis, we regulated the expression of circNFIB and L1CAM in PDAC cell lines. Western blot results showed that overexpression of circNFIB significantly increased phosphorylated ERK levels, whereas knockdown of L1CAM abolished this effect (Figure 6D, Figure S4B, Figure S6H, Figure S6O).

Next, we added the ERK inhibitor SCH772984, BVD-523, and LY3214996 to block ERK phosphorylation in our experiments. Our comparative results demonstrated that all three inhibitors significantly reduced the PNI capacity of pancreatic cancer cells. However, SCH772984 exhibited the most potent inhibitory effect (Figure S4C). Western blot analysis confirmed that SCH772984 significantly reduced phosphorylated ERK levels (Figure 6E, Figure S4D, Figure S6I, Figure S6P). We then conducted Matrigel/DRG model assays, DRG matrix assays, Transwell assays, and neural adhesion assay. The results showed that overexpression of circNFIB promoted PNI in PDAC, whereas the addition of SCH772984 reversed this effect (Figure 6F-I, Figure S4E-H). These results demonstrate that circNFIB promotes PNI in PDAC by activating the ERK/MAPK signaling pathway.

### RBMS1 Promotes the Biogenesis of circNFIB in PDAC by Regulating Intron Back-Splicing of NFIB Pre-mRNA

**Figure 7:**
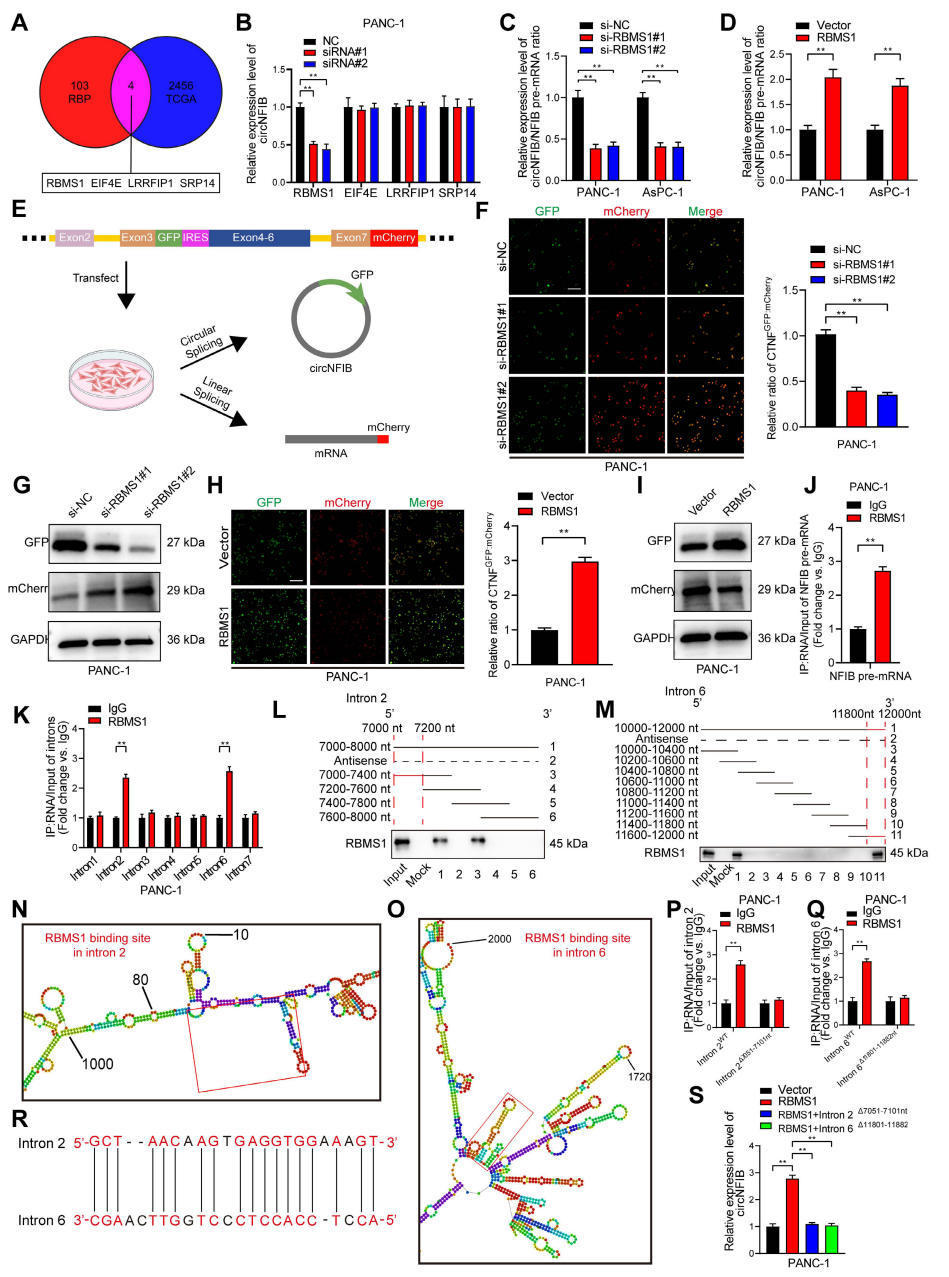
** RBMS1 Binds to Flanking Introns of the circNFIB Splicing Exon and Promotes circNFIB Biogenesis in PDAC.** (A) Schematic diagram screening four RBPs that may influence circRNA formation in PDAC. (B) qRT-PCR analysis of circNFIB expression following knockdown of RBMS1, EIF4E, LRRFIP1, and SRP14. (C, D) Relative circNFIB/NFIB pre-mRNA expression ratio upon RBMS1 knockdown (C) or overexpression (D). (E) Schematic diagram of the dual-fluorescence reporter model. (F) Representative images and quantification of circNFIB and NFIB mRNA expression in RBMS1-knockdown PANC-1 cells. Scale bar = 50 μm. (G) Western blot analysis of GFP and mCherry expression in RBMS1-knockdown PANC-1 cells. (H) Representative images and quantification of circNFIB and NFIB mRNA expression in RBMS1-overexpressing PANC-1 cells. Scale bar = 50 μm. (I) Western blot analysis of GFP and mCherry expression in RBMS1-overexpressing PANC-1 cells. (J) RIP assay investigating the interaction between RBMS1 and NFIB pre-mRNA in PANC-1 cells. (K) RIP assay investigating the interaction between RBMS1 and intron1-7 in PANC-1 cells. (L, M) RNA pull-down assay using truncated sequences of intron 2 (L) and intron 6 (M) to determine the region required for RBMS1 interaction. (N, O) Schematic representation of predicted RBMS1 binding sites on intron 2 (N) and intron 6 (O). (P, Q) RIP assay investigating the interaction between RBMS1 and site-directed mutants of intron 2 (7051-7101 nt) and intron 6 (11801-11882 nt). (R) Schematic representation of the reverse complementary sequence of introns 2 and 6. (S) Expression of circNFIB in PANC-1 cells following deletion of RBMS1 binding sites in introns 2 and 6. Statistical differences in B, C, F, and S were analyzed using one-way ANOVA followed by Dunnett's test, while D, H, J, K, P, and Q were analyzed using a two-tailed Student's t-test. Error bars represent SD from three independent experiments. *p < 0.05, **p < 0.01.

As previously discussed, circRNA biogenesis originates from back-splicing of pre-mRNA 31. Using CircInteractome, we predicted RBPs that may interact with circNFIB, including EIF4A3 32. However, after knocking down EIF4A3, the expression of circNFIB in PDAC cells did not show significant changes (Figure S5A). A study on circRNA biogenesis summarized 103 RBPs known to affect circRNA synthesis 33. We explored the expression levels of these RBPs in PDAC tissues from the Cancer Genome Atlas Program (TCGA) database, identifying four RBPs with high expression in PDAC (logFC ≥ 2 and *p* < 0.01) (Figure 7A). Next, we knocked down these four RBPs in the PANC-1 cell line and found that knockdown of RBMS1 significantly reduced circNFIB expression (Figure 7B).

To investigate whether RBMS1 promotes circNFIB biogenesis by regulating the back-splicing of NFIB pre-mRNA, we conducted qRT-PCR analysis in PDAC cells with knockdown or overexpression of RBMS1. The results showed that the expression of circNFIB significantly decreased or increased, while the level of NFIB pre-mRNA remained unchanged (Figure S5B-E). Additionally, knocking down or overexpressing RBMS1 altered the ratio of circNFIB to NFIB mRNA (Figure 7C-D). These results suggest that RBMS1 regulates circNFIB expression by promoting its biogenesis rather than affecting the expression of the parent gene, NFIB. To verify this, we constructed a dual-fluorescence reporter system that detects circNFIB biogenesis through GFP translation mediated by IRES, while mCherry expression was used to monitor linear NFIB pre-mRNA production (Figure 7E) 13, 34. The experimental results showed that knocking down or overexpressing RBMS1 significantly lowered or increased the GFP/mCherry ratio, respectively. Similarly, we observed consistent results in Western blotting (Figure 7F-I, Figure S5F-I, Figure S6J-K, Figure S6Q-R), confirming that RBMS1 promotes circNFIB biogenesis in PDAC.

As reported in previous studies, RNA-binding proteins induce circRNA biogenesis by directly binding to adjacent intronic regions of pre-mRNA to promote back-splicing 9. To determine whether RBMS1 binds directly to NFIB pre-mRNA, we performed RIP experiments (Figure 7J, Figure S5J). We designed primers targeting the exonic-adjacent intronic regions of circNFIB, and the results showed that RBMS1 bound significantly more to introns 2 and 6 than to other distal regions of NFIB pre-mRNA (Figure 7K). Further RNA pull-down experiments using probes designed for the truncated sequences of introns 2 and 6 confirmed RBMS1 binding to these specific regions (Figure 7L-M, Figure S5K-M). Using the catRAPID tool (http://service.tartaglialab.com/page/catrapid_omics2_group), we predicted specific binding sites of RBMS1 within NFIB pre-mRNA (Figure 7N-O). Mutation of introns 2 (7051-7101 nt) and 6 (11801-11882 nt) disrupted the binding of RBMS1 to NFIB pre-mRNA (Figure 7P-Q, Figure S5N-O). Additionally, we identified complementary sequences near introns 2 and 6 that interact with RBMS1 (Figure 7R).

Finally, using CRISPR/Cas9 technology, we deleted the intronic segments (7051-7101 nt of intron 2 and 11801-11882 nt of intron 6) from NFIB pre-mRNA to verify whether RBMS1's binding affects circNFIB biogenesis in PDAC. The results showed that deletion of these segments significantly reduced RBMS1-mediated upregulation of circNFIB in PDAC (Figure 7S). In conclusion, our findings demonstrate that RBMS1 promotes the biogenesis of circNFIB in PDAC by binding to specific intronic regions of NFIB pre-mRNA (intron 2 [7051-7101 nt] and intron 6 [11801-11882 nt]).

### CircNFIB as a Potential Therapeutic Target to Alleviate the Antitumor Effects of SCH772984

**Figure 8:**
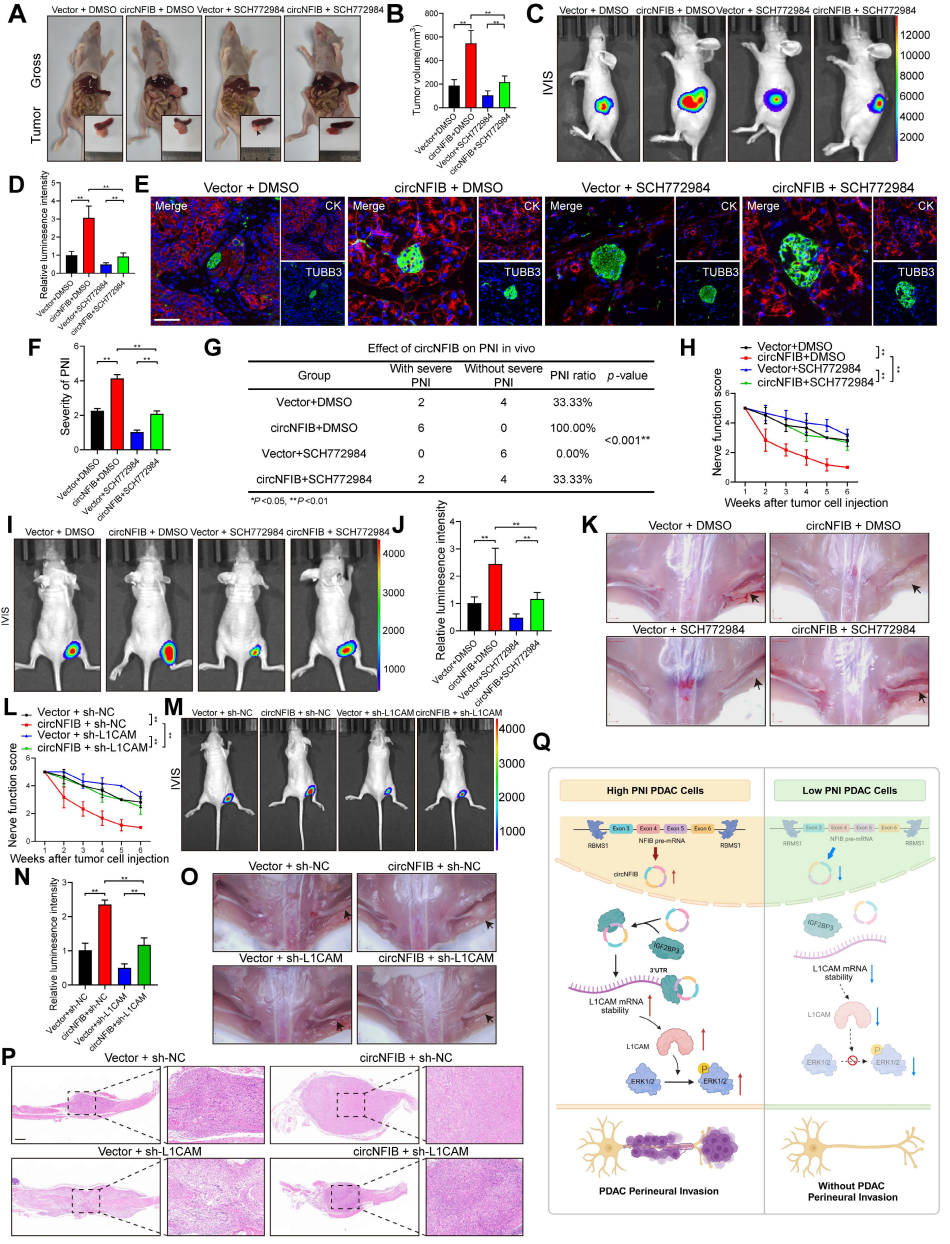
** CircNFIB as a Potential Therapeutic Target and Its Attenuation of the In Vivo Antitumor Effect of SCH772984.** (A-D) Representative images and quantification of pancreatic tumors harvested from the orthotopic xenograft model (A, B), and corresponding IVIS images and luminescence quantification (C, D) (n = 6 nude mice per group). (E, F) Representative images and quantification of multiplex immunohistochemical staining for CK and TUBB3 in tumor tissues from the orthotopic xenograft model. Scale bar = 100 μm. (G) PNI incidence rate in tumor tissues from the orthotopic xenograft model. (H) Nerve function scoring in nude mice implanted with PANC-1 cells. (I-K) IVIS images and luminescence quantification (I, J), and representative images of harvested tumors (K) in the in vivo sciatic nerve invasion model (n = 6 nude mice per group). (L) Nerve function scoring in nude mice implanted with PANC-1 cells. (M-P) IVIS images and luminescence quantification (M, N), representative images of harvested tumors (O), and H&E staining of sciatic nerves (P) in the in vivo sciatic nerve invasion model (n = 6 nude mice per group). (Q) Schematic illustration of the proposed mechanism. Statistical analysis: Panels B, D, F, H, J, L, and N were analyzed using one-way ANOVA followed by Dunnett's post hoc test. Panel G was analyzed using the χ² test. Error bars represent SD from three independent experiments. *p < 0.05, **p < 0.01.

SCH772984 is a novel ERK kinase inhibitor that specifically inhibits the activation of ERK1/2, effectively blocking the ERK/MAPK pathway 35. It has been proven to have significant inhibitory effects on various tumors. To investigate the influence of circNFIB on SCH772984, we conducted in vivo experiments with four groups: Vector + DMSO, circNFIB + DMSO, Vector + SCH772984, and circNFIB + SCH772984. In an orthotopic xenograft model of PDAC, SCH772984 suppressed tumor growth and PNI, while overexpression of circNFIB was able to rescue this inhibitory effect (Figure 8A-G). Similarly, in a mouse sciatic nerve injection model, SCH772984 significantly reduced the ability of PDAC cells to invade along the sciatic nerve. However, overexpression of circNFIB could also restore this effect, counteracting SCH772984's inhibition (Figure 8H-K). These findings demonstrate that circNFIB can activate the phosphorylation of ERK in vivo, thereby mitigating the inhibitory effects of SCH772984 on PNI in PDAC. This suggests that targeting circNFIB could be a promising therapeutic strategy to overcome the limitations of ERK inhibitors in PDAC treatment.

Given the critical role of L1CAM in pancreatic cancer PNI, we established a PANC-1 sciatic nerve injection model with circNFIB overexpression and/or lentiviral-mediated L1CAM knockdown (sh-L1CAM) to evaluate the therapeutic potential of L1CAM as a target for PNI intervention. Nerve function scoring revealed that circNFIB overexpression significantly accelerated hind limb dysfunction in mice, whereas L1CAM knockdown effectively alleviated nerve function decline (Figure 8L). IVIS imaging demonstrated that circNFIB overexpression promoted tumor spread along the sciatic nerve, while this effect was markedly attenuated by L1CAM silencing. Quantification of luminescence intensity further confirmed that the PNI capacity in the circNFIB + sh-L1CAM group was significantly lower than in the circNFIB + sh-NC group (Figure 8M-N). Gross anatomical examination of the sciatic nerve region revealed restricted tumor extension in the L1CAM knockdown group. H&E staining of sciatic nerve sections further verified that L1CAM silencing significantly reduced both the severity and frequency of PNI (Figure 8O-P). Collectively, these results indicate that lentiviral knockdown of L1CAM reverses the PNI-promoting effect of circNFIB, highlighting the clinical significance of the circNFIB/L1CAM axis as a potential therapeutic target in PDAC.

## Discussion

CircRNA, due to its unique closed-loop structure, exhibits significantly stronger structural stability compared to linear RNA 36. This characteristic makes it a highly promising candidate as a diagnostic and prognostic biomarker for PDAC. Recent studies have elucidated the crucial role of circRNA in influencing the progression of PDAC. For instance, circARFGEF2 promotes the activation of JAK2, mediating lymphatic metastasis in KRAS-mutant PDAC cells 13. Despite these advancements, PNI remains one of the leading causes of high recurrence rates and poor survival in PDAC patients. Therefore, a deeper understanding of the key circRNAs driving PNI in PDAC, along with their molecular mechanisms, is of significant clinical importance for improving patient prognosis.

In this study, we identified for the first time the circRNA, circNFIB, which is strongly associated with the occurrence of PNI in PDAC and poor prognosis. Through a series of in vivo and in vitro experiments, we validated its role in promoting PNI in PDAC. Furthermore, we discovered that circNFIB promotes PNI in PDAC by activating the L1CAM/MAPK signaling pathway. Additionally, we revealed the crucial role of RBMS1 in the biogenesis of circNFIB, thus enriching our understanding of the regulatory role of RBPs in circRNA generation.

Although previous studies have suggested that circNFIB exhibits growth and metastasis inhibition in certain cancer types, such as intrahepatic cholangiocarcinoma and breast cancer, where circNFIB regulates specific signaling pathways to suppress tumor cell proliferation and migration, this study is the first to reveal the different role of circNFIB in PDAC 37, 38. Specifically, we found that circNFIB promotes PDAC neural invasion by stabilizing L1CAM mRNA and activating the ERK/MAPK signaling pathway. This difference may be related to the distinct molecular mechanisms of various tumor types, the tumor microenvironment, and the interactions between circNFIB and other molecules 39, 40. This suggests that the function of circNFIB is tumor-type dependent, where it may regulate different targets in different tumor contexts, leading to diverse outcomes. This not only deepens our understanding of the complex functions of circRNAs in tumor biology but also provides new theoretical support for future individualized treatment strategies based on circRNAs.

L1 cell adhesion molecule (L1CAM) is a transmembrane glycoprotein initially identified in the nervous system, where it plays a key role in neural development 41. However, increasing evidence suggests that L1CAM is also a critical factor in various malignancies, particularly in promoting tumor cell migration, invasion, and neural invasion 42. Overexpression of L1CAM in PDAC has been linked to PNI and poor prognosis 43, 44. Previous studies have highlighted the association between L1CAM and multiple signaling pathways, such as the PI3K/AKT and MAPK pathways 29, 30, 45. However, the mechanism by which L1CAM regulates PNI in PDAC remains unclear. In this study, we performed RNA-seq to identify differential expression between circNFIB-overexpressing and control PDAC cells and found that the MAPK signaling pathway was significantly enriched. Previous studies have shown that various PNI-related molecules can influence PDAC invasion by regulating the MAPK signaling pathway 46, but the relationship between L1CAM and the MAPK pathway in PNI has not been thoroughly explored. Our study demonstrates that circNFIB, as an upstream regulator of L1CAM, amplifies its pro-invasive effects through the MAPK pathway. Furthermore, we discovered that circNFIB promotes ERK phosphorylation (p-ERK), thereby enhancing PNI in PDAC. The use of the ERK phosphorylation inhibitor SCH772984 significantly reversed the promoting effect of circNFIB overexpression on PNI. These findings suggest that circNFIB regulates PDAC PNI via the ERK/MAPK signaling axis, providing a novel molecular regulatory pathway and expanding our understanding of the mechanisms underlying PNI in PDAC.

CircRNAs exert their biological functions through various mechanisms, with one well-studied role being their function as miRNA sponges that regulate cellular activities. CircRNAs can also interact with RBPs to modulate cellular functions 47. In this study, we identified the circNFIB-binding protein IGF2BP3 through RNA pulldown combined with mass spectrometry and further confirmed that IGF2BP3 enhances the stability of L1CAM mRNA in a circNFIB-dependent manner. IGF2BP3 is an RNA-binding protein that is closely associated with the onset of various tumors 28, 48. IGF2BP3 has been widely reported to alter the stability of mRNA by binding to the 3' untranslated region (UTR) of the mRNA 27. This study further expands our understanding of IGF2BP3 in RNA metabolism and tumor progression. Despite IGF2BP3's well-established role in interacting with various RNAs and carrying out multiple functions, its precise regulatory mechanisms remain unclear and require further investigation.

RBMS1 has been reported to be involved in regulating mRNA stability and translation 49, 50, making it a potential therapeutic target in cancer treatment. However, this study is the first to demonstrate that RBMS1 promotes the biogenesis of circNFIB by binding to the flanking introns of its precursor RNA, thereby expanding the functional understanding of RBMS1 in non-coding RNA regulation. This discovery broadens the role of RBMS1 in RNA metabolism and highlights its significance in the regulation of non-coding RNA generation. It is noteworthy that whether RBMS1 broadly regulates the biogenesis of other circRNAs and its functional role in different cancer types remains to be further explored. However, whether RBMS1 is involved in the regulation of a broader range of circRNA biogenesis, the functions of the circNFIB-mediated L1CAM/MAPK axis beyond PNI, and whether this regulatory axis can serve as a new strategy for targeted therapy in PDAC, still require further research.

## Conclusions

Our study demonstrates that circNFIB is upregulated in PNI in PDAC and plays a crucial role in the occurrence of PNI through the circNFIB-L1CAM/MAPK axis (Figure 8Q). Furthermore, we discovered that RBMS1 regulates the reverse splicing of introns in NFIB pre-mRNA, promoting the biosynthesis of circNFIB in PDAC. This research expands our understanding of circRNA in tumor biology, provides new insights into the molecular mechanisms of PNI in PDAC, and lays the theoretical foundation for circRNA as a potential therapeutic target for PDAC.

## Supplementary Material

Supplementary figures, tables and methods.

## Figures and Tables

**Table 1:** Association between circNFIB expression levels and clinicopathologic characteristics of PDAC patients.

Characteristics	No. of cases	circNFIB expression level		
		Low	High	*p* ^a^
Total cases	96	48	48	
Gender				0.682
Male	52	25	27	
Female	44	23	21	
Age				0.399
≤60	36	16	20	
>60	60	32	28	
Differentiation				0.422
Poor	4	2	2	
Moderate	32	19	13	
Well	60	27	33	
TNM stage				0.219
Stage I	47	21	26	
Stage II	38	23	15	
Stage III	11	4	7	
PNI				
Without PNI	34	34	0	<0.001^**^
PNI	62	14	48	

Abbreviations: No. of cases = number of cases. ^a^Chi-square test, *p < 0.05, **p < 0.01.

## Abbreviations

3'-UTR: 3' untranslated region
circRNAs: circular RNAs
DEGs: differentially expressed genes
DRG: dorsal root ganglia
FBS: fetal bovine serum
FISH: fluorescence in situ hybridization
GEO: Gene Expression Omnibus
Ig: immunoglobulin
IGF2BP3: Insulin-like growth factor II mRNA-binding protein 3
JNK: c-Jun N-terminal kinase
KEGG: Kyoto Encyclopedia of Genes and Genomes
L1CAM: L1 cell adhesion molecule
MAPK: mitogen-activated protein kinase
mIHC: multiplex immunohistochemistry
ORF: open reading frame
OS: overall survival
PDAC: pancreatic ductal adenocarcinoma
PNI: perineural invasion
p-ERK: ERK phosphorylation
pre-mRNAs: precursor mRNAs
RIP: RNA Immunoprecipitation
RBP: RNA-binding protein
siRNA: small interfering RNA
SD: standard deviation
TCGA: the Cancer Genome Atlas Program
