# Supplementary material for: RBMS1-mediates the biogenesis of circNFIB promotes perineural invasion of pancreatic ductal adenocarcinoma via the L1CAM/MAPK pathway
Source: Theranostics. 2025 Aug 16;15(17):9261–78. doi: 10.7150/thno.112753 (PMC12439468; doi:10.7150/thno.112753)
Supplement: Supplementary file 1 — Supplementary figures, tables and methods. [file thnov15p9261s1.pdf]

Supplementary data

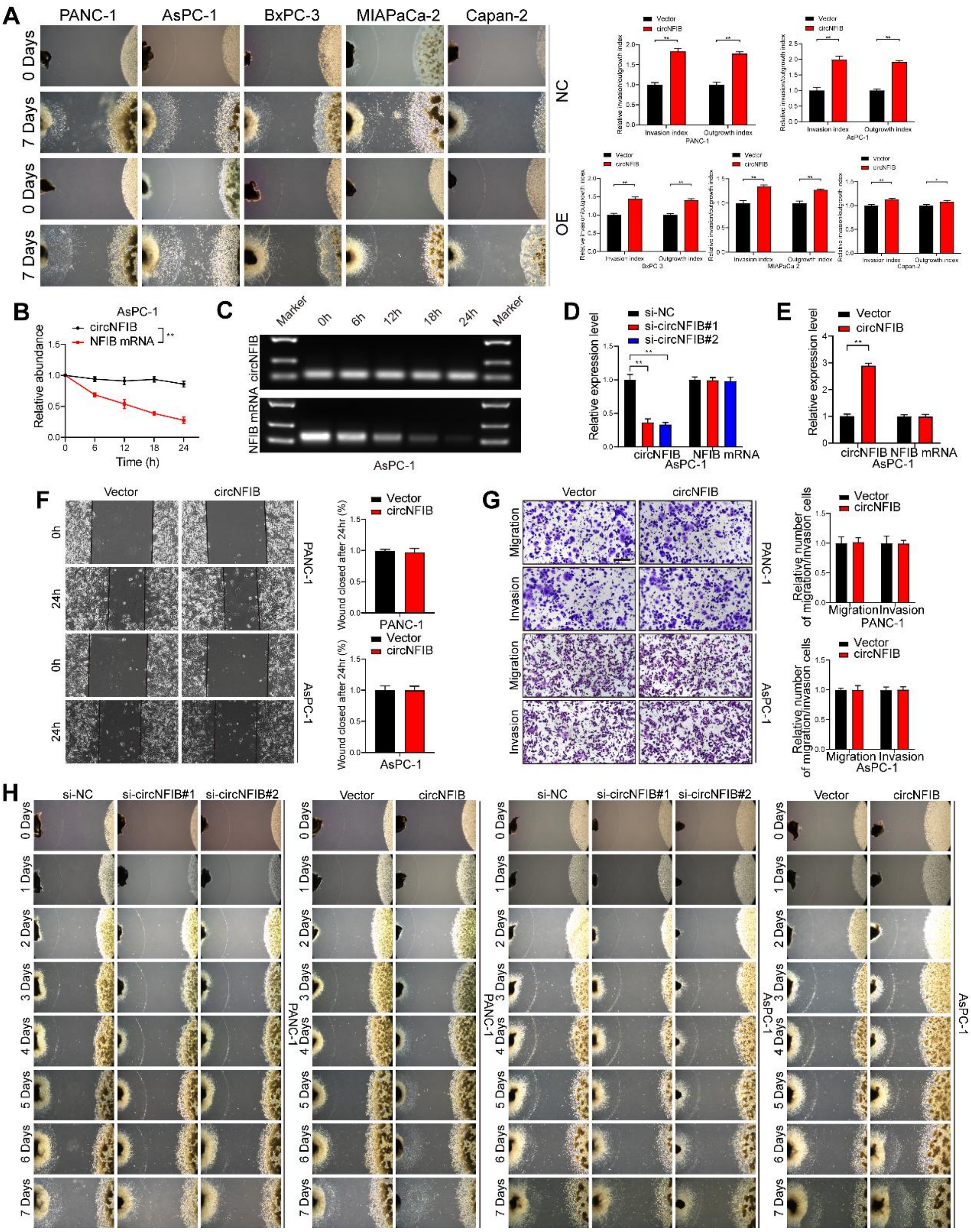

Figure S1. CircNFIB promotes PNI of PDAC in vitro. (A) Representative images and quantification of the Matrigel/DRG co-culture model using five pancreatic cancer cell lines, showing their nerve invasion and outgrowth capacities. (B, C) Stability of circNFIB and NFIB mRNA in AsPC-1 cells at the indicated time points was assessed using actinomycin D treatment (B) and agarose gel

electrophoresis (C). (D, E) qRT-PCR analysis of circNFIB and NFIB expression levels in AsPC-1 cells with circNFIB knockdown (D), circNFIB overexpression (E), and their respective control groups. (F) Representative images and quantification of wound healing assays in PANC-1 and AsPC-1 cells following circNFIB overexpression. (G) Representative images and quantification of Transwell migration and Matrigel invasion assays in PANC-1 and AsPC-1 cells overexpressing circNFIB. Scale bar = 100 μm. (H) Representative images and quantification of the Matrigel/DRG model using the indicated PANC-1 and AsPC-1 cells, evaluating their nerve invasion and neurite-associated growth. Statistical significance was determined using two-tailed Student's t-tests in (A, B, E, F, and G) and one-way ANOVA followed by Dunnett's post hoc test in (D). Error bars represent the standard deviation from three independent experiments. *$p < 0.05$, **$p < 0.01$.

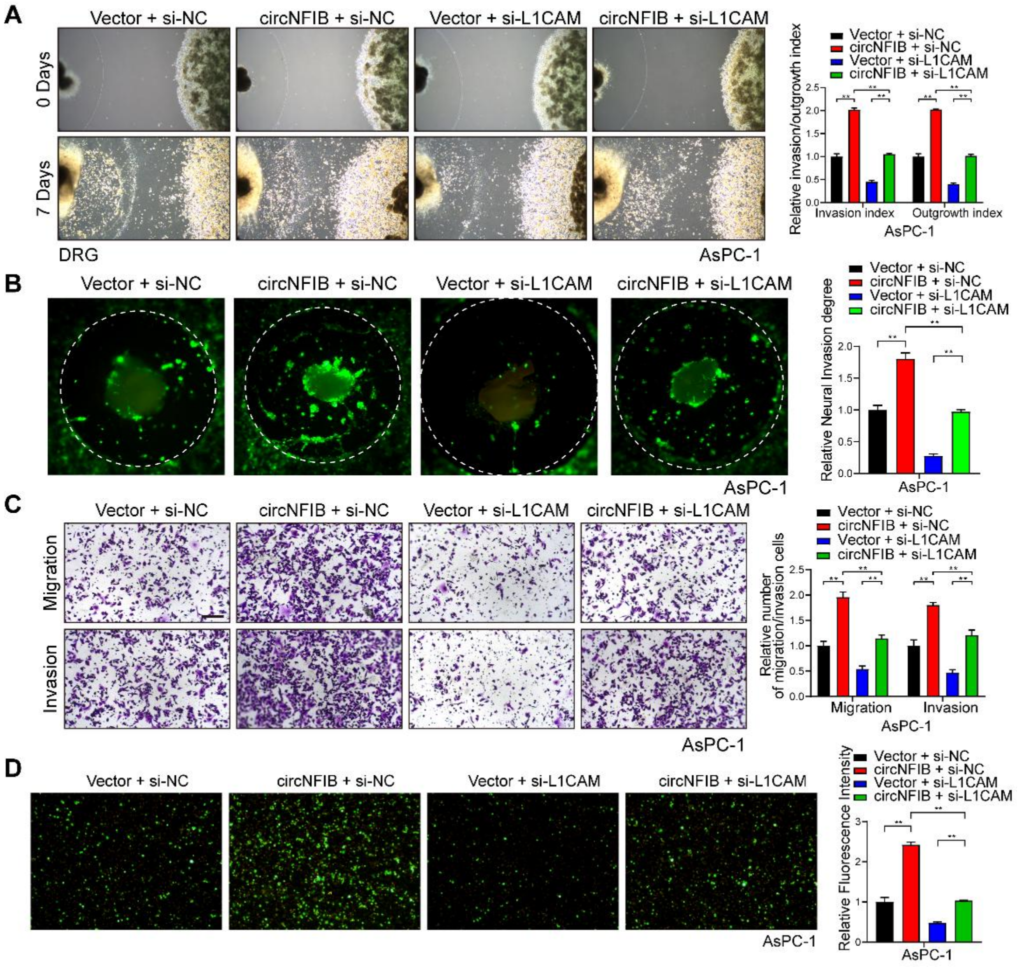

Figure S2. CircNFIB promotes PNI of PDAC by upregulating L1CAM expression. (A) Representative images and quantification of the Matrigel/DRG model using the indicated AsPC-1 cells, evaluating their nerve invasion and neurite-associated growth capacity. (B) Representative DRG matrix images and quantification of nerve invasion capacity in the indicated AsPC-1 cells. (C) Representative images and quantification of Transwell migration and Matrigel invasion assays in the indicated AsPC-1 cells. Scale bar = 100 μm. (D) Representative images and quantification of the neural adhesion assay in the indicated AsPC-1 cells, showing their cell–nerve adhesion capacity. One-way ANOVA followed by Dunnett's post hoc test was used for statistical analysis in (A, B, C, and D). Error bars represent the standard deviation from three independent experiments. *$p < 0.05$, **$p < 0.01$.

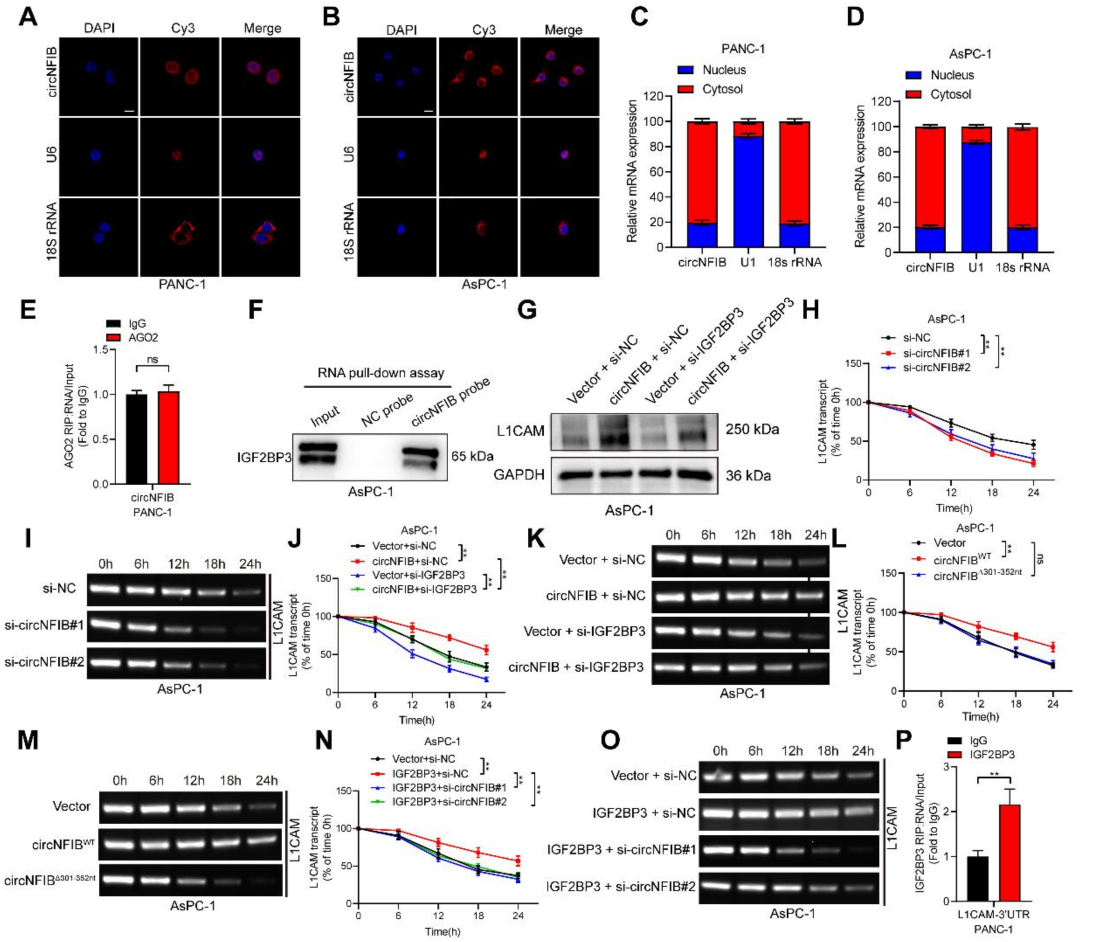

Figure S3. CircNFIB interacts with IGF2BP3 to promote L1CAM mRNA stability in AsPC-1 cells. (A, B) FISH analysis showing the subcellular localization of circNFIB. Scale bar = 20 μm. (C, D) Subcellular fractionation assays confirming the localization of circNFIB in PANC-1 (C) and AsPC-1 (D) cells. U1 was used as the nuclear control, and 18S rRNA was used as the cytoplasmic control. (E) RIP assay investigating the interaction between AGO2 and circNFIB in PANC-1 cells. (F) Western blotting analysis of the interaction between circNFIB and IGF2BP3 in AsPC-1 cells. (G) Western blotting analysis of L1CAM expression levels in the indicated AsPC-1 cells. (H–O) Actinomycin D treatment followed by quantification and representative agarose gel electrophoresis images of L1CAM mRNA in the indicated AsPC-1 cells. (P) RIP assay examining the interaction between IGF2BP3 and the 3'-UTR of L1CAM mRNA. Statistical significance in (H, J, L, and N) was determined using one-way ANOVA followed by Dunnett's post hoc test; (E) and (P) were analyzed by two-tailed Student's t-

test; (C) and (D) were analyzed using the χ^2 test. Error bars represent the standard deviation from three independent experiments. *$p < 0.05$, **$p < 0.01$.

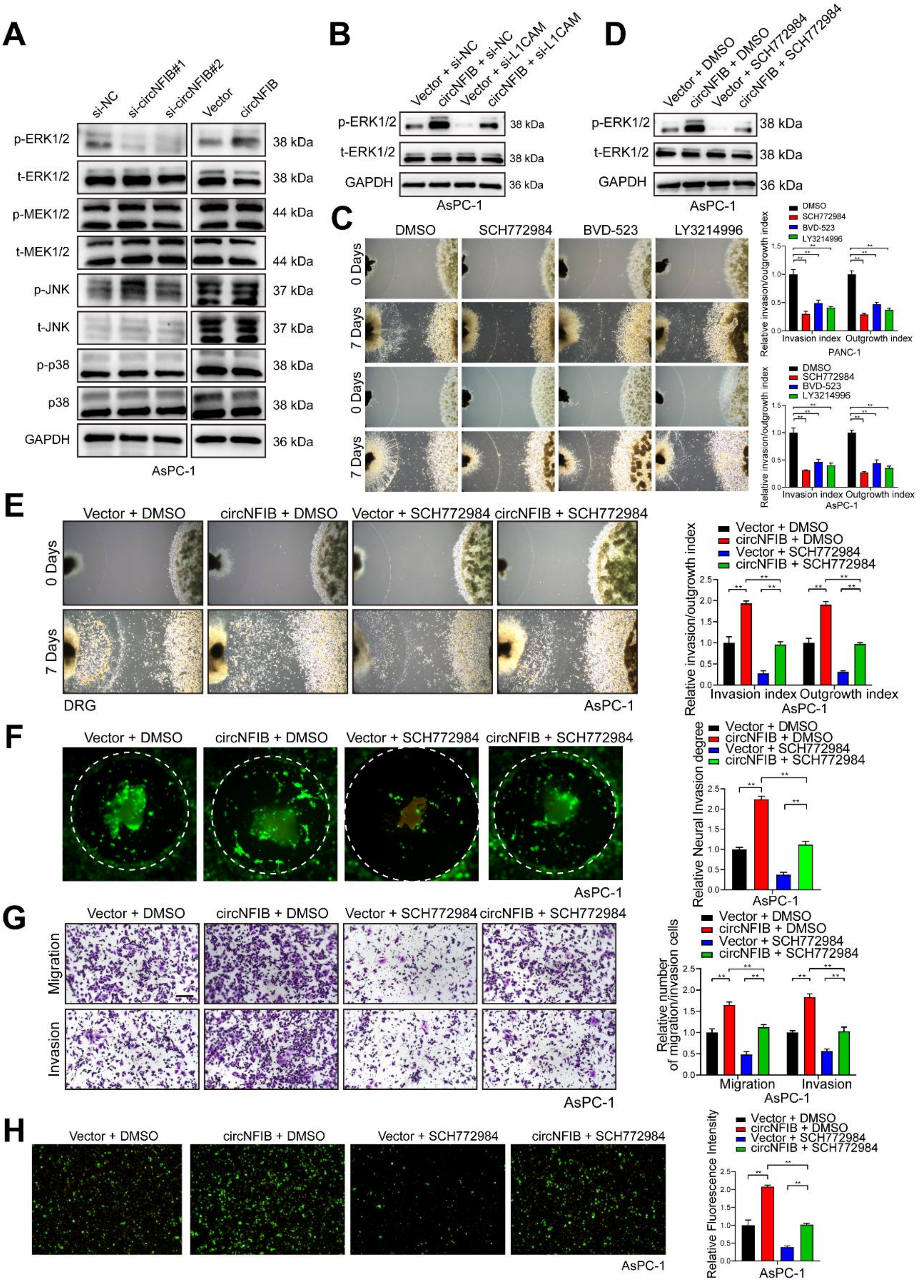

Figure S4. CircNFIB promotes PNI by activating the ERK/MAPK axis in PDAC. (A) Western blotting analysis of MAPK signaling pathway–related proteins in AsPC-1 cells with circNFIB knockdown or overexpression. (B) Western blotting analysis of p-ERK and t-ERK levels in AsPC-1

cells following circNFIB and L1CAM modulation. (C) Representative images and quantification of the Matrigel/DRG model using the indicated PANC-1 cells, evaluating their nerve invasion and neurite-associated growth capacity. (D) Western blotting analysis of p-ERK and t-ERK levels in AsPC-1 cells after treatment with SCH772984. (E) Representative images and quantification of the Matrigel/DRG model using the indicated AsPC-1 cells, evaluating their nerve invasion and neurite-associated growth capacity. (F) Representative DRG matrix images and quantification of nerve invasion capacity in the indicated AsPC-1 cells. (G) Representative images and quantification of Transwell migration and Matrigel invasion assays in the indicated AsPC-1 cells. Scale bar = 100 μm. (H) Representative images and quantification of the neural adhesion assay in the indicated AsPC-1 cells, showing their cell–nerve adhesion capacity. Statistical significance in (C, E, F, G, and H) was determined using one-way ANOVA followed by Dunnett's post hoc test. Error bars represent the standard deviation from three independent experiments. *$p < 0.05$, **$p < 0.01$.

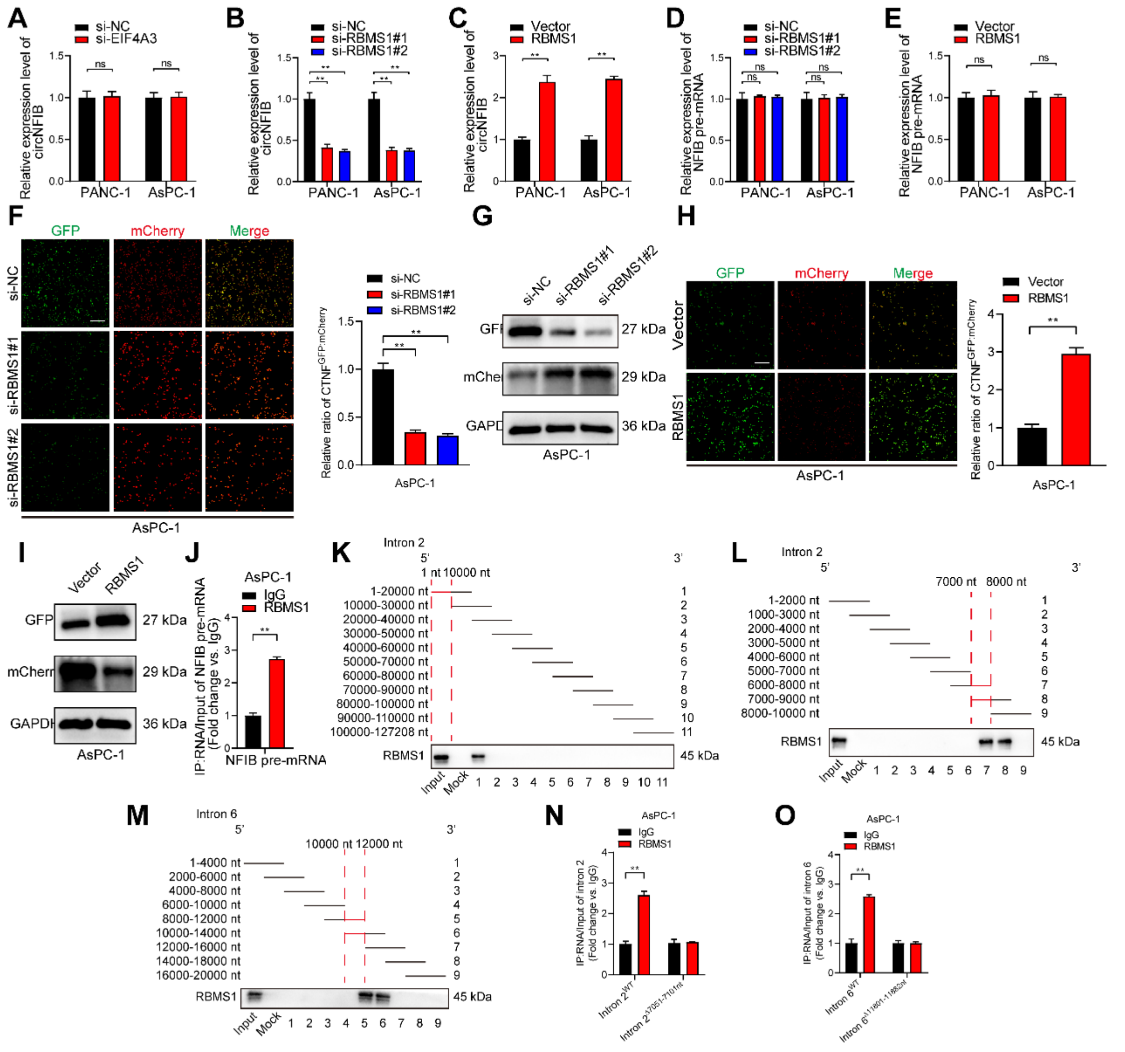

Figure S5. RBMS1 Regulates the Biogenesis of circNFIB in PDAC. (A) qRT-PCR analysis of circNFIB expression after EIF4A3 knockdown. (B, C) qRT-PCR analysis of circNFIB expression in PDAC cells with RBMS1 knockdown (B) or overexpression (C). (D, E) qRT-PCR analysis of NFIB pre-mRNA expression in PDAC cells with RBMS1 knockdown (D) or overexpression (E). (F) Representative images and quantification of circNFIB and NFIB mRNA expression in AsPC-1 cells with RBMS1 knockdown. Scale bar = 50 μm. (G) Western blot analysis of GFP and mCherry expression in AsPC-1 cells with RBMS1 knockdown. (H) Representative images and quantification of circNFIB and NFIB mRNA expression in AsPC-1 cells with RBMS1 overexpression. Scale bar = 50 μm. (I) Western blot analysis of GFP and mCherry expression in AsPC-1 cells with RBMS1 overexpression. (J) RIP assay

investigating the interaction between RBMS1 and NFIB pre-mRNA in AsPC-1 cells. (K-M) RNA pull-down assay using truncated sequences of intron 2 (K, L) and intron 6 (M) to identify the regions required for RBMS1 interaction. (N, O) RIP assay investigating the interaction between RBMS1 and site-directed mutated sequences of intron 2 (7051-7101 nt) (N) and intron 6 (11801-11882 nt) (O). Statistical differences in B, D, and F were analyzed using one-way ANOVA with Dunnett's test; A, C, E, H, J, N, and O were analyzed using a two-tailed Student's t-test. Error bars represent standard deviation from three independent experiments. *$p < 0.05$, **$p < 0.01$.

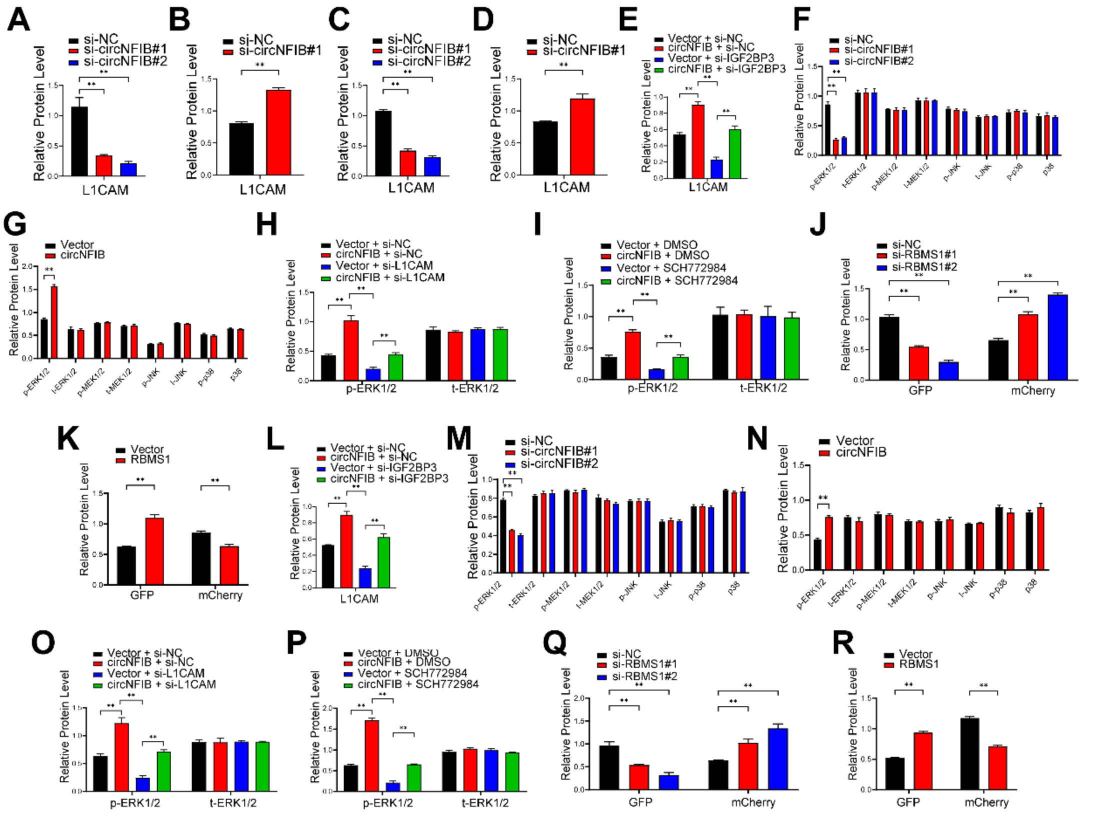

Figure S6. Statistical analysis of Western blot experiments evaluating the role of circNFIB in PDAC cells. Data represent mean ± standard deviation from three independent experiments. Statistical significance was determined using one-way ANOVA followed by Dunnett's post hoc test or two-tailed Student's t-test as appropriate. *$p < 0.05$, **$p < 0.01$.

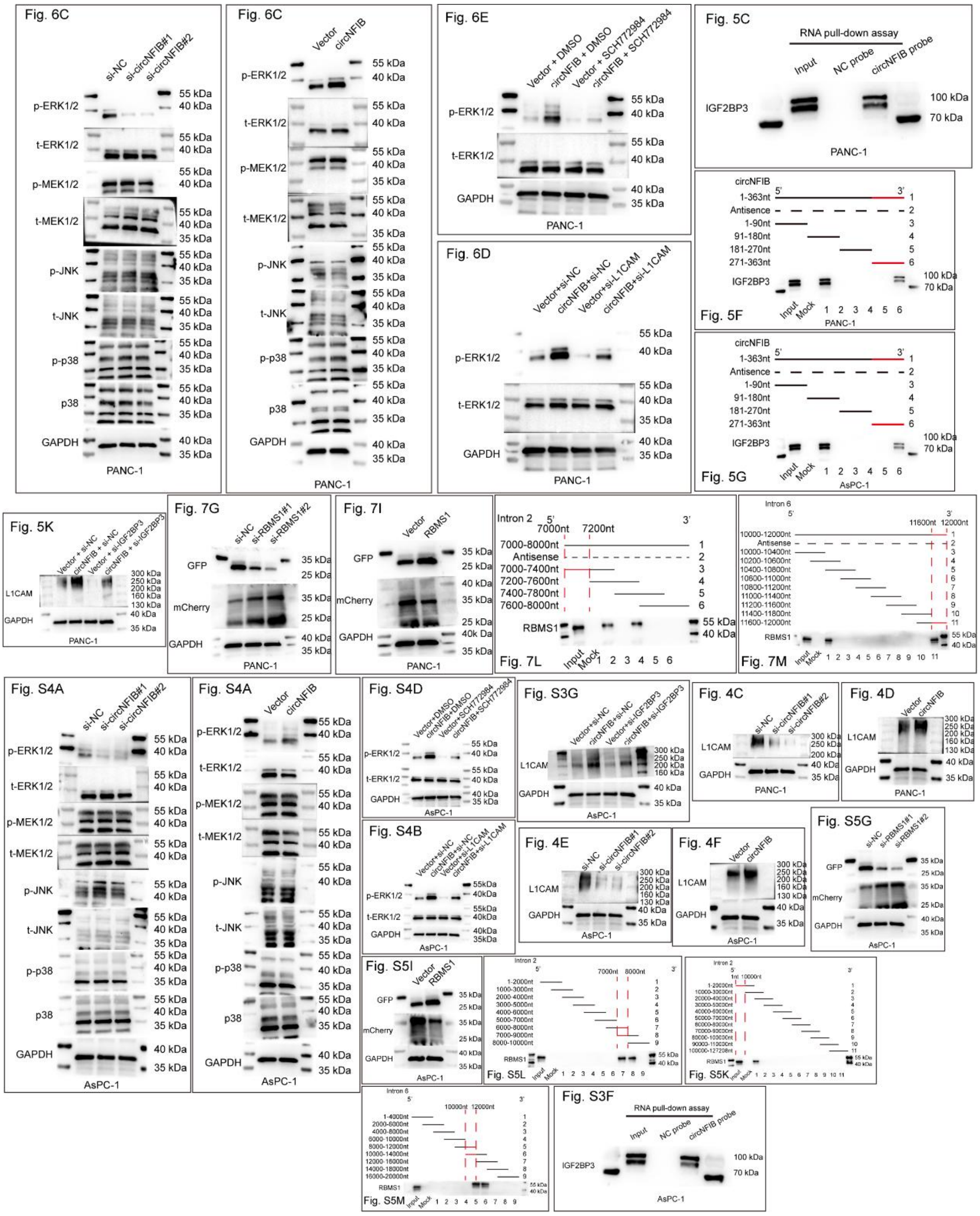

83

84 **Figure S7. Full uncropped original pictures for western blot assays.**

85

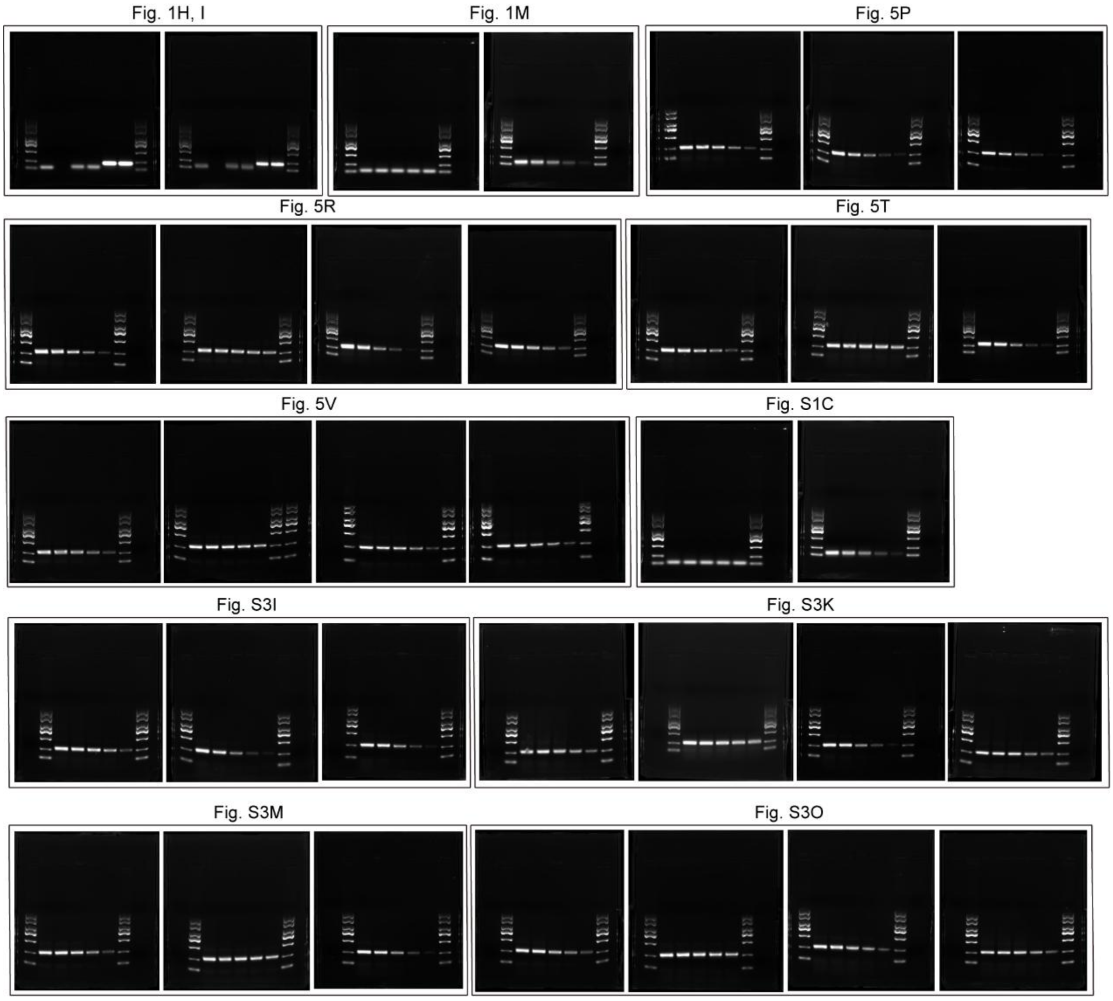

Figure S8. Full uncropped original pictures for Gels.

TableS1. Univariate and multivariate analyses of Overall Survival (OS) for circNFIB expression.

Variables	Univariate analysis			Multivariate analysis		
	**HR**	**95%CI**	p^a	**HR**	**95%CI**	p^a
Age (<60 vs. ≥60)	0.85	0.51-1.42	0.530			
Gender (Male vs. Female)	1.01	0.61-1.65	0.985			
circNFIB expression (High vs. Low)	1.72	1.04-2.86	$\mathbf{0.036}^{*}$	1.72	1.04-2.86	$\mathbf{0.036}^{*}$

Abbreviations: HR = hazard ratio; 95% CI =95% confidence interval. [a]Cox regression analysis, $*p < 0.05$, $**p < 0.01$.

Table S2. Primer and probes used in the experiments.

Gene	Sequence (5’-3’)	Application
circNFIB	F: CGAAAGAGATCAAGATTCTGGAC	qRT-PCR
	R: CCTGGGTTATGGGCGTTCT	
NFIB	F: GCTGTGTCTTATCCAATCCCG	qRT-PCR
	R: TGCCTTTGAACAGGATCACCA	
GAPDH	F: CACATCGCTCAGACACCATG	qRT-PCR
	R: TGACGGTGCCATGGAATTTG	
U1	F: CAGGGGAGATAACGTGACCA	qRT-PCR
	R: GGGAAAAGCACGGACACAG	
18S rRNA	F: GCGGCGGAAAATAGCCTTTG	qRT-PCR
	R: GATCACACGTTCCACCTCATC	
SEMA3D	F: TCAGAGCACTACTGGCTCAAT	qRT-PCR
	R: ATCGGAGGTACTGCCTTCTTG	
L1CAM	F: CCGACAACCACTCAGACTACA	qRT-PCR
	R: CCGGAGGTCAATGGGTTCC	
MUC1	F: TGCCGCCGAAAGAACTACG	qRT-PCR
	R: TGGGGTACTCGCTCATAGGAT	
TRKA	F: AACCTCACCATCGTGAAGAGT	qRT-PCR
	R: TGAAGGAGAGATTCAGGCGAC	
TRKB	F: ACCCGAAACAAACTGACGAGT	qRT-PCR
	R: AGCATGTAAATGGATTGCCCA	
CX3CR1	F: AGTGTCACCGACATTTACCTCC	qRT-PCR
	R: AAGGCGGTAGTGAATTTGCAC	
EGFR	F: AGGCACGAGTAACAAGCTCAC	qRT-PCR
	R: ATGAGGACATAACCAGCCACC	
NGF	F: TGTGGGTTGGGGATAAGACCA	qRT-PCR
	R: GCTGTCAACGGGATTTGGGT	
ARTN	F: GACGAGCTGGTGCGTTTC	qRT-PCR

	R: ATGAAGGAGACCGCTTCGTA	
PTN	F: GGAGCTGAGTGCAAGCAAAC	qRT-PCR
	R: CTCGCTTCAGACTTCCAGTTC	
si-circNFIB#1	Sense: GAGAUCAAGAUUCUGGACAAUTT	siRNA
	Antisense: AUUGUCCAGAAUCUUGAUCUCTT	
si-circNFIB#2	Sense: GAUCAAGAUUCUGGACAAUCATT	siRNA
	Antisense: UGAUUGUCCAGAAUCUUGAUCTT	
si-IGF2BP3	Sense: GGUGCUGGAUAGUUUACUATT	siRNA
	Antisense: UAGUAAACUAUCCAGCACCTT	
si-RBMS1#1	Sense: CCAUAUACCUUUCAACCUAAUTT	siRNA
	Antisense: AUUAGGUUGAAAGGUAUAUGGTT	
si-RBMS1#2	Sense: CCACAGAACCUUUAUUGUGUATT	siRNA
	Antisense: UACACAAUAAAGGUUCUGUGG<br>TT	
si-EIF4E#1	Sense: CCAAAGAUAGUGAUUGGUUAUTT	siRNA
	Antisense: AUAACCAAUCACUAUCUUUGGTT	
si-EIF4E#2	Sense: CCGACUACAGAAGAGGAGAAATT	siRNA
	Antisense: UUUCUCCUCUUCUGUAGUCG<br>GTT	
si-LRRFIP1#1	Sense: CGCACAGUACAGAAGUAGGUATT	siRNA
	Antisense: UACCUACUUCUGUACUGUGC<br>GTT	
si-LRRFIP1#2	Sense: CCAAGUACAGUAGACACUCAATT	siRNA
	Antisense: UUGAGUGUCUACUGUACUUG<br>GTT	
si-SRP14#1	Sense: CCUCCUUAGAGCUAACAUGGATT	siRNA
	Antisense: UCCAUGUUAGCUCUAAGGAG<br>GTT	
si-SRP14#2	Sense: GUCGAACCAAACCCAUUCCAATT	siRNA

	Antisense: UUGGAAUGGGUUUGGUUCGA CTT	
circNFIB	ACTTCC+TGATTG+TCCAGAA+TCTTGATCTCTTTCGTG CCAT	Pull-down
circNFIB	ACTTCC+TGATTG+TCCAGAA+TCTTGATCTCTTTCGTG CCAT	FISH
U6	TTTGCGTGTCATCCTTGCG	FISH
18S rRNA	CTTCCTTGGATGTGGTAGCCGTTTC	FISH

Table S3. Antibodies used in the experiments

Product	Source	No. of Catalogue
**Primary antibody:**		
**Western blot:**		
anti-L1CAM	Abcam	ab270455
anti-GAPDH	Proteintech	60004-1-Ig
anti-IGF2BP3	Proteintech	14642-1-AP
anti-p-ERK1/2	Cell Signaling Technology	4370T
anti-t-ERK1/2	Cell Signaling Technology	4695T
anti-p-MEK1/2	Cell Signaling Technology	2338T
anti-t-MEK1/2	Cell Signaling Technology	8727T
anti-p-JNK	Proteintech	80024-1-RR
anti-t-JNK	Proteintech	51153-1-AP
anti-p-p38	Cell Signaling Technology	4511T
anti-p38	Cell Signaling Technology	9212S
anti-GFP	Abcam	ab183735
anti-mCherry	Abcam	ab125096
anti-RBMS1	Proteintech	11061-2-AP
**Immunofluorescence:**		
anti-IGF2BP3	Proteintech	14642-1-AP
**Immunohistochemistry：**		
anti-pan-CK	Abcam	ab7753
anti-beta III Tubulin	Abcam	ab18207
**Immunoprecipitation:**		
anti-AGO2	Proteintech	67934-1-Ig
anti-IGF2BP3	Proteintech	14642-1-AP
anti-RBMS1	Proteintech	11061-2-AP
**Secondary antibody:**		
**Western blot:**		
anti-rabbit IgG-HRP	Beijing Ray Antibody Biotech	RM3002

anti-mouse IgG-HRP	Beijing Ray Antibody Biotech	RM3001
**Immunofluorescence:**		
Alexa Fluor 488 (Goat Anti-Rabbit IgG H&L)	Abcam	ab150077

Supplemental methods

Western Blot assay

Cell samples were lysed using RIPA lysis buffer (CWBIO, China) supplemented with 1% phosphatase inhibitor and 1% protease inhibitor. The lysates were centrifuged at 12,000 rpm for 30 min at 4 °C, and the supernatants were collected. Protein concentrations were quantified using a BCA protein assay kit (CWBIO, China). Proteins were separated by SDS-PAGE gel electrophoresis (Epizyme, China) and subsequently transferred onto PVDF membranes (MERCK, Germany). The membranes were blocked with a rapid protein-free blocking buffer (Epizyme, China) and incubated overnight at 4 °C with primary antibodies (Table S3). The following day, the membranes were washed with TBST buffer and incubated with secondary antibodies at room temperature for 1 h. After three additional washes with TBST, protein expression levels were detected using an enhanced ECL chemiluminescent substrate (Tanon, China).

RNA Pull-Down assay

First, biotin-labeled circNFIB probes and negative control (NC) probes were incubated overnight at 4 °C with PDAC cell lysates. On the following day, the incubated lysates were mixed with magnetic beads (Thermo Scientific, USA) and further incubated at 4 °C for 3 h. Subsequently, proteins bound to circNFIB were eluted and stained using the Pierce Silver Staining Kit (Thermo Scientific, USA). Differential bands were identified through mass spectrometry analysis.

Cell culture and transfection

All cell lines used in this study were obtained from the American Type Culture Collection (ATCC, USA), and their identities were validated by short tandem repeat (STR) analysis. The hTERT-HPNE, PANC-1,

and MiaPaCa-2 cells were cultured in DMEM medium (VivaCell, China), while AsPC-1 and BxPC-3 cells were cultured in RPMI 1640 medium (VivaCell, China), and Capan-2 cells were cultured in McCoy's 5A medium (VivaCell, China). The media were supplemented with 10% fetal bovine serum (VivaCell, China) and 1% penicillin-streptomycin solution (NCM, USA), and cells were maintained in a humidified incubator at 37 °C with 5% CO_2.

All siRNAs and overexpression plasmids were synthesized by GenePharma (Shanghai, China) or IGE (Guangzhou, China). Transfections were performed using Lipofectamine™ 3000 transfection reagent (Invitrogen, USA) according to the manufacturer’s protocol. The sequences of all siRNAs are listed in Table S2.

Orthotopic Xenograft Model

Nude mice were anesthetized with inhaled pentobarbital, and a skin incision was made along the left costal margin to expose the pancreas. A total of 1×10^6 transfected PDAC cells were injected into the pancreas, followed by suturing of the incision. Six weeks later, tumors were imaged using an IVIS imaging system. In situ tumor specimens were then collected for multiplex immunohistochemistry (mIHC) to evaluate the severity of perineural invasion (PNI) in the primary tumors.

RNA extraction and quantitative real-time PCR (qRT-PCR)

RNA from pancreatic cancer patient surgical specimens or PDAC cell lines was extracted using TRIzol reagent (Takara Bio, Japan). The RNA concentration was measured using a Nanodrop One spectrophotometer (Thermo Scientific, USA). After reverse transcription of RNA into cDNA using the

Hiscript III reverse transcriptase kit (Vazyme, China), quantitative real-time PCR (qRT-PCR) was performed using the ChamQ™ Universal SYBR qPCR Master Mix (Vazyme, China) on a CFX96™ real-time system (Bio-Rad, USA). The sequences of all primers are listed in Table S2.

RNase R Treatment

In the experimental group, 1 μg of RNA was treated with 2 U of RNase R (Epicentre Technologies, USA), while the control group received 1 μg of RNA mixed with an equal volume of DEPC water. The expression levels of circNFIB and NFIB mRNA were then analyzed by qRT-PCR, with GAPDH used as the internal control.

Actinomycin D Assay

PDAC cells were seeded into a six-well tissue culture plate at a density of 1×10^5 cells per well. The following day, the cells were treated with 2 μg/mL of actinomycin D (Sigma, USA) at time points of 6 h, 12 h, 18 h, and 24 h. RNA was extracted, and the expression levels of circNFIB, NFIB, and L1CAM were analyzed by qRT-PCR.

Fluorescence In Situ Hybridization (FISH) Assay

PDAC cells (2×10^4 cells) were seeded into a confocal dish. The following day, the cells were fixed with 4% paraformaldehyde for 20 min, then treated with 0.5% Triton X-100 for 10 min to permeabilize the cells. After three washes with PBS, the cells were incubated with 200 μL of pre-hybridization solution at 37 °C for 30 min. Next, the cells were incubated overnight at 37 °C with 2.5 μL of 20 μM circNFIB FISH probe (GenePharma, China), followed by DAPI staining. Images were captured using a confocal

fluorescence microscope (Carl Zeiss AG, Germany).

Nuclear and Cytoplasmic Extraction

The cytoplasmic and nuclear fractions were separated using the PARIS Kit (Thermo Scientific, USA). PDAC cells were lysed in pre-chilled cell fractionation buffer for 10 min. The lysates were then centrifuged at 500 g for 3 min at 4 °C, and the supernatant was collected as the cytoplasmic fraction, while the pellet represented the nuclear fraction. U1 and 18S rRNA were used as internal controls for the nuclear and cytoplasmic fractions, respectively. The distribution of circNFIB in the nucleus and cytoplasm was detected by qRT-PCR, with primer sequences listed in Table S2.

Silver Staining

The protein samples obtained from the RNA pull-down assay were collected and separated by 10% SDS-PAGE gel electrophoresis. The gel was then stained and visualized using a silver staining kit (Thermo Scientific, USA).

Dual Luciferase Assay

Luciferase activity was measured using the luciferase reporter assay system (Promega, USA) following the manufacturer's instructions. Cell lysates were prepared to detect luciferase activity, and readings were taken using the MK3 microplate reader (Thermo, USA). Renilla luciferase was used as an internal control for normalization.

Immunofluorescence (IF) Assay

PDAC cells (2×10^4 cells) were seeded into a confocal dish. The following day, the cells were fixed with 4% paraformaldehyde for 20 min, then permeabilized with 0.5% Triton X-100 for 10 min. After blocking with blocking buffer at 37 °C for 1 h, the cells were incubated with primary antibodies overnight at 4 °C. Afterward, they were stained with fluorescent secondary antibodies and DAPI. Images were captured using a confocal fluorescence microscope (Carl Zeiss AG, Germany).

Multiplex Immunohistochemistry (mIHC)

Pancreatic cancer tumor tissue sections were stained using the PANO 4-plex IHC kit (Panovue, China). Paraffin sections were first deparaffinized twice with xylene, followed by rehydration through an ethanol gradient. Antigen retrieval was performed under high temperature using EDTA. The sections were then blocked with goat serum (Golden Bridge Biological Technology, China) for 15 min, followed by incubation with primary antibodies for 1 h. Afterward, the sections were incubated with horseradish peroxidase (HRP)-conjugated secondary antibodies for 30 min, and then stained with substrate solution for 15 min. EDTA antigen retrieval was repeated between each staining step. Finally, the nuclei were stained with DAPI for 15 min, and images were captured using a confocal fluorescence microscope (Carl Zeiss AG, Germany).

Dual-Color Fluorescence Reporter Assay

The dual-color fluorescent reporter plasmids were designed and synthesized by IGE (Guangzhou, China). GFP with a FLAG tag and mCherry with an HA tag, both of which include a nuclear localization signal, were used. Cells (2×10^5) were transfected with the plasmids and cultured in a six-well plate. After 2 days, 2×10^4 cells were seeded onto a confocal dish. Images were captured using a confocal fluorescence

microscope (Carl Zeiss AG, Germany).

Neural Adhesion Assay

Primary DRG neurons were isolated from neonatal rats and seeded into 6-well plates at equal density. After 24 h, 1×10^5 GFP-labeled PDAC cells with indicated circNFIB and/or L1CAM knockdown or overexpression were added to each well and co-cultured for 1 hour at 37 °C. Following incubation, non-adherent cells were removed by PBS washing. GFP-positive tumor cells adhered to DRG neurons were quantified using a fluorescence plate reader and verified by fluorescence microscopy.
